# Luminescence-Based Strategies for Detecting β-Lactamase Activity: A Review of the Last Decade

**DOI:** 10.3390/life16020250

**Published:** 2026-02-02

**Authors:** Michał Jakub Korytkowski, Anna Baraniak, Alicja Boryło, Paweł Rudnicki-Velasquez

**Affiliations:** 1 Department of Pharmaceutical Microbiology and Laboratory Diagnostics, National Medicines Institute, Chełmska 30/34, 00-725 Warsaw, Poland; m.korytkowski@nil.gov.pl (M.J.K.); a.baraniak@nil.gov.pl (A.B.); 2 Department of Chemistry and Radiochemistry of Environment, Faculty of Chemistry, University of Gdansk, Wita Stwosza 63 Str., 80-308 Gdansk, Poland; alicja.borylo@ug.edu.pl; 3 Department of Falsified Medicines and Medical Devices, National Medicines Institute, Chełmska 30/34, 00-725 Warsaw, Poland

**Keywords:** β-lactamases, β-lactam resistance, ESBL, carbapenemases, luminescence, bioluminescence, chemiluminescence, fluorescence, rapid diagnostics

## Abstract

Rapid detection of β-lactamase activity is becoming increasingly important as β-lactam resistance spreads at an alarming rate and conventional diagnostics often require several hours to deliver actionable results. Over the past decade, methods based on luminescence, bioluminescence, chemiluminescence, and fluorescence have become powerful tools for the functional assessment of resistance resulting from β-lactamase activity. These approaches provide highly sensitive, activity-based readouts, often within minutes, and frequently rely on simple optical instrumentation. In this review, we summarize recent developments in luminescent probe design between 2015 and 2025, with emphasis on reaction mechanisms, analytical performance, and the ability of these systems to discriminate between different β-lactamases, including narrow-spectrum enzymes, AmpC, ESBL, and carbapenemases. We also discuss their applications in bacterial cultures, clinical isolates, complex biological matrices and, in some cases, in vivo models. While luminescent assays are not yet positioned to replace standard susceptibility testing, they offer a practical and increasingly robust complement to culture-based and molecular methods. The emerging trends highlighted here, such as self-immobilizing fluorogenic probes, chemiluminescent relay systems, nanomaterial-based sensors and AI-assisted mobile platforms, suggest that luminescent β-lactamase detection could play a meaningful role in future rapid diagnostics and resistance surveillance.

## 1. Introduction

Despite general advances in many fields of medicine, the therapy of bacterial infections is facing major challenges and remains one of the main public health problems, not only in areas with limited resources, but also in developed countries. Several factors contribute to this situation, including antimicrobial resistance (AMR) of pathogenic bacteria, an ageing population, changes in social habits, international travel, population dynamics and climate change [1,2]. The emergence and spread of AMR pathogens, driven by increasing global antibiotic consumption, leads to the occurrence of incurable bacterial illnesses and could undermine decades of progress in the treatment of infectious diseases [3,4,5,6]. The real consequences of this crisis are evidenced by the number of AMR-related deaths, which reached nearly five million in 2019 and are expected to rise to ten million annually by 2050 [5,7]. Besides the human aspect, there is also an economic one, associated with the costs of treatment, the number of additional days of hospitalization and reduced productivity. To counteract the catastrophic vision of a “post-antibiotic era” various national and global AMR combat strategies have been implemented, such as the development of new medicines and vaccines, the rational use of antibiotics, infection prevention and control, the improvement of existing diagnostics, and the invention of new, faster methods for detecting AMR mechanisms [7,8,9,10].

Among the many available antimicrobial drugs, β-lactams are the safest, largest and most diverse group of antibiotics, used to treat almost all types of infections of various aetiologies. All these contribute to β-lactams accounting for more than half of all antibiotics used worldwide in human medicine [11,12]. Therefore, the development and rapid spread of β-lactam resistance among pathogens, mainly Gram-negative bacteria, is currently the most serious clinical and epidemiological AMR issue [13]. Among the various types of such resistance, the main mechanism in Enterobacterales and non-fermenting bacilli is the production of β-lactamases, so the detection of β-lactamase activity is applied in the diagnostics of this type of resistance [14]. In general, methods of β-lactamase detection can be divided into three groups: tests based on phenotypic detection, identification of the β-lactamase-encoding gene, and assays based on β-lactamase hydrolysis. The first group includes the diffusion method, the dilution method (determination of the minimum inhibitory concentration, MIC, of antibiotic), and automatic detection. All of them are mainly used in clinical diagnostics and enable indirect detection of β-lactamase production and antibiotic resistance based on susceptibility test results, but they require appropriate environmental conditions, have a long detection time and relatively low specificity and sensitivity. Genetic methods, mainly involving polymerase chain reaction (PCR), isothermal amplification using loop-mediated isothermal amplification and DNA microarrays, are generally characterised by high sensitivity and specificity and shorter detection times compared to phenotypic tests, but they are more expensive, require the use of specialised equipment and may produce false positive results, which to a certain extent limit their clinical applications. In turn, hydrolysis-based methods for detecting β-lactamases include acidimetry, iodometry, spectrophotometry (UV–Vis), mass spectrometry-based approaches, lateral flow immunoassays, and luminescent probes. The first three techniques are based on the properties of penicilloic acid, which is formed during the hydrolysis of a β-lactam by a β-lactamase. The other three methods detect β-lactamases by identifying antibiotic substrates or their physically and biologically hydrolysed products [15]. The latter method using luminescent probes deserves special attention due to its enormous potential for development as a new method for detecting β-lactamases, considering its high detection efficiency, high sensitivity, and very short detection time.

This review summarises recent advances (2015–2025) in luminescence-based strategies for detecting β-lactam resistance due to β-lactamase activity. It focuses on probe design, detection mechanisms and analytical performance across different luminescent platforms. We also discuss the current limitations that prevent routine clinical implementation and outline where further progress is needed.

## 2. Methods

This review focuses on the development over the last decade of luminescence-based methods, including fluorescence, chemiluminescence and bioluminescence, used to detect bacterial resistance caused by β-lactamase production. In July 2025, a structured literature search was carried out in three major databases: PubMed, Scopus, and Web of Science. Search strings combined terms related to luminescence (“luminescence,” “fluorescence,” “photoluminescence,” “chemiluminescence,” “bioluminescence”) with those describing antibiotic resistance (“antibiotic resistance,” “antimicrobial resistance,” “resistant bacteria,” “β-lactamase,” “β-lactam resistance,” “carbapenemase”). Each query was adjusted to the syntax requirements of the respective database. To minimize omissions, reference lists of the initially selected papers were also screened manually. This snowballing step helped identify additional studies describing emerging luminescent probes or less common resistance mechanisms that might not have appeared in keyword-based searches. The screening included only original, peer-reviewed research articles published in English that used a luminescence-based method to detect, visualise, or characterise a defined antimicrobial-resistance mechanisms. Priority was given to studies involving fluorescent probes, chemiluminescent substrates, bioluminescent reporters, or hybrid luminescent systems designed to recognise resistance-associated enzymes or resistant bacterial strains. Review papers, conference abstracts, patents, and studies not directly related to luminescence-based detection of resistance were excluded. Patents were not considered further, as they generally lack the level of experimental detail, standardized performance metrics, and peer-reviewed validation required for critical comparison of analytical sensitivity, specificity, and practical applicability. For each eligible publication, key information was extracted, including the type of used luminescent mechanism, the molecular nature of the sensing system (e.g., enzyme, fluorophore, nanoparticle), and analytical parameters such as sensitivity, specificity, and limits of detection (LODs). All collected information was synthesised into a narrative summary, supported by concise comparative tables that group the publications according to luminescence type and the resistance mechanism targeted.

## 3. Brief Overview of β-Lactams and β-Lactamases

β-Lactams, like other antimicrobial agents, have been continuously modified since their introduction into treatment in order to improve their properties, including potency, spectrum of activity, pharmacokinetic features, and safety profile, as well as to counteract developing resistance [16]. A common characteristic of the β-lactam structure is the presence of a ring containing three carbon atoms and one nitrogen atom and depending on its modification, different groups of these antibiotics are distinguished [17]. Generally, they include penicillins (natural penicillins, aminopenicillins, carboxypenicillins and ureidopenicillins), in which β-lactam ring is fused to thiazolidine ring; cephalosporins (divided into five generations), where a six-membered dihydrotiazin is attached; carbapenems, where the bicyclic system is completed by a five-membered pyrroline; and monobactams (monocyclic systems) [18,19]. The antibacterial action of β-lactams is based on the steric similarity of the β-lactam ring to the terminal D-alanine-D-alanine group of the peptidoglycan pentapeptide. Transpeptidases, known as penicillin-binding proteins, which are involved in the final stage of peptidoglycan synthesis, “mistakenly” recognize β-lactams as a substrate during cell wall synthesis, which results in the blocking of their activity, loss of viability, and lysis of bacterial cells [19,20].

As with other antimicrobial agents, the widespread use of β-lactams has led to the emergence and spread of resistance to this group of antibiotics. The production of β-lactamases, enzymes capable of opening the β-lactam ring and rendering the antibiotic inactive, is the most common and important resistance mechanism in Gram-negative bacteria [19]. There are two main classification schemes for β-lactamases, the Ambler sequence homology-based system and the Bush-Jacoby-Medeiros activity-based system [21,22]. The first scheme divides β-lactamases into four classes: A, B, C, and D, where enzymes belonging to classes A, C, and D have serine in their active site (serine β-lactamases, SBLs), while class B uses zinc cations as cofactors for hydrolysis reactions (metallo-β-lactamases, MBLs). The second model distinguishes four (from 1 to 4) functional groups according to substrate hydrolysis profiles (penicillin, cephalosporin, extended-spectrum cephalosporin, carbapenem) and inhibitor profiles (inhibition by β-lactam inhibitors such as clavulanate and tazobactam or EDTA). Group 1 contains enzymes commonly referred to as cephalosporinases AmpC (e.g., ACC-1, ACT-1, ADC-1, CMY-1, FOX-1, MIR-1, MOX-1 and PAC-1), which are poorly inhibited or uninhibited by clavulanic acid. Group 2 is the largest and most diverse in structure, substrate preferences, and sensitivity to β-lactam inhibitors. It consists of narrow-spectrum β-lactamases (e.g., LAP-1, PC1-1 and OXA-10), broad-spectrum β-lactamases (e.g., CAE-1, CARB-1, CfxA-1, ROB-1, SCO-1, SHV-1, TEM-1 and OXA-1), extended-spectrum β-lactamases, ESBLs (e.g., BEL-1, CARB-10, CfxA-2, CTX-M-1, SFO-1, GES-1, KLUC-3, KPC-12, OHIO-1, PER-1, PME-1, SHV-2, TEM-3, TLA-1, TLA2-1, VEB-1 and OXA-2), as well as carbapenemases (e.g., BIC-1, BKC-1, FLC-1, FRI-1, GES-4, GPC-1, KPC-1, VCC-1 and OXA-48). Group 3 includes MBL enzymes (e.g., DIM-1, FIM-1, GIM-1, IMP-1, NDM-1, SIM-1, SPM-1, TMB-1 and VIM-1), which are inhibited by EDTA and are uninhibited by β-lactam inhibitors. Group 4 consists of only a few enzymes identified long ago and poorly characterised; in the latest version of the functional classification system, this group has been excluded. Both β-lactamase classification schemes correlate well with each other; all enzymes forming functional group 1 constitute structural class C, group 2 contains β-lactamases of classes A and D, while group 3 corresponds to class B [21,22,23]. For clarity, the relationships between the Bush–Jacoby–Medeiros functional classification and the Ambler molecular classification, together with their key catalytic and inhibition features, are summarized in Table 1.

Over the past decades, resistance caused by the production of β-lactamases by Gram-negative bacteria has been increasing at an alarming rate. To date, over 12,000 different β-lactamases have been identified [23]. Among them, acquired β-lactamases are of particular concern, as their genes are located on mobile genetic elements (e.g., plasmids), enabling the spread of this type of resistance between different bacterial cells, including different species, through horizontal gene transfer [13,24,25]. This concerns mainly ESBLs, which represent a major problem among Enterobacterales by conferring resistance to all penicillins, cephalosporins (except cephamycins), and monobactams. However, the most dangerous resistance mechanism in Gram-negative rods is the production of carbapenemases, enzymes capable of inactivating all β-lactams, including carbapenems, agents regarded as drugs of last resort [20,26,27].

## 4. Luminescence Mechanisms: Principles and Classification

### 4.1. Background of Luminescence

Luminescence is the emission of light by a substance through mechanisms that do not rely on heat. In simple terms, it is photon release from electronically excited molecules produced by chemical reactions, biochemical processes, or absorbed radiation [28,29]. Contrary to incandescence, it does not require high temperatures and can occur under mild physiological conditions, thus making these processes ideal for biological and microbiological applications.

**Figure 1:**
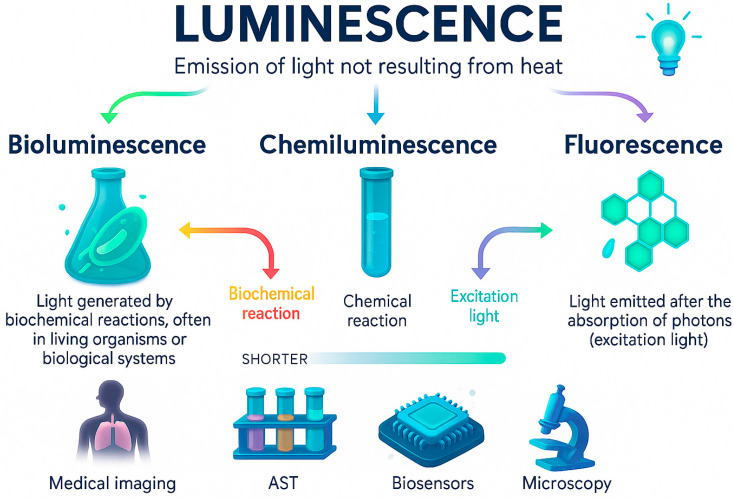
The overview of main forms of luminescence.

In biomedical research, luminescent readouts offer sensitive, non-invasive ways to monitor biological activity. Among the different forms of luminescence, bioluminescence, chemiluminescence, and fluorescence (Figure 1) are the most widely used in microbiology and antibiotic resistance studies. For example, chemiluminescence and bioluminescence have been exploited to evaluate bacterial viability under antibiotic exposure by comparing light emission from treated and untreated cultures [30,31]. Fluorescence-based platforms, including nanoparticle- and quantum dot–assisted systems, have enabled rapid and low-cost assessment of antibiotic susceptibility in microorganisms [32,33]. Recent studies and reviews highlight the expanding use of optical, including luminescence-based, biosensors for the detection of antibiotic residues as well as resistance-associated genes in environmental samples [34,35,36].

### 4.2. Bioluminescence

Bioluminescence is a naturally occurring form of light emission that arises from the enzymatic oxidation of a small-molecule substrate (luciferin) by a luciferase enzyme. The reaction typically requires molecular oxygen and, depending on the system, may also require cofactors such as ATP and Mg^2+^ [37,38,39]. Light is produced when luciferin is oxidized to an excited intermediate (e.g., oxyluciferin in fireflies), which releases a photon as it relaxes back to the ground state [38]. In bacteria, the best-known example is the marine species *Aliivibrio fischeri* (formerly *Vibrio fischeri*), whose luxCDABE operon encodes both the luciferase subunits (luxAB) and the enzymes that synthesize its long-chain aldehyde substrate (luxCDE). This flavin-dependent monooxygenase system oxidizes FMNH_2_ together with the aldehyde, emitting blue-green light at approximately 490–495 nm [40,41,42,43]. Because the lux operon includes both the enzyme and its substrate-generating operon machinery, it enables autonomous light production without the need to add an external luciferin. The full lux cassette has also been successfully expressed in non-bacterial hosts, including mammalian and plant cells, highlighting its versatility [42,43]. In molecular biology, bioluminescent reporters such as luxAB, firefly luciferase (luc), and *Gaussia* luciferase (Gluc) are routinely used to track gene expression, viability, and metabolic activity in real time. Engineered into *Escherichia coli*, these reporters allow fast and sensitive readouts of bacterial responses during antibiotic susceptibility testing (AST), detecting changes in metabolic activity long before conventional growth measurements do [32,44,45,46]. *Gaussia* luciferase, in particular, has been used as a secreted reporter in pathogenic *E. coli* during murine infection studies, with luminescence correlating closely with colony-forming unit (CFU) counts in tissue-cage fluid and plasma. This enables both in vivo imaging and quantitative ex vivo monitoring of bacterial burden [47,48,49]. Bioluminescent readouts are also used to monitor enzymatic activities, including β-lactamase hydrolysis, supporting rapid identification of resistant bacterial phenotypes. In vivo, bioluminescence imaging enables non-invasive tracking of infection progression and therapeutic response, although it is subject to limitations such as oxygen dependency, limited tissue penetration of emitted light, and potential metabolic burden associated with reporter expression [50,51,52,53].

Overall, bioluminescence relies on luciferase-driven oxidation of luciferin, producing light either autonomously (lux systems) or with the addition of an external substrate. This makes it a sensitive and flexible tool in microbiology, including diagnostics and AMR research, particularly where non-invasive or real-time readouts are advantageous.

### 4.3. Chemiluminescence

Chemiluminescence is the emission of light generated directly by a chemical reaction, without the need for external illumination; in many systems the light-emitting step itself is non-enzymatic, even if enzymes may participate upstream in probe activation. Most chemiluminescent reactions involve a redox process that produces an electronically excited intermediate; light is released when the intermediate relaxes back to the ground state. A well-known example is the luminol reaction, in which luminol is oxidized by hydrogen peroxide in the presence of a catalyst such as hemoglobin or Cu^2+^, producing blue light with a peak around 425 nm [44,54,55,56]. Chemiluminescent readouts are widely used in clinical diagnostics, immunoassays, microbiology, and environmental monitoring. In bacterial detection, they have been applied to measure toxins, metabolic markers, and AMR mechanisms. A major application is chemiluminescent enzyme immunoassays (CLEIA), which rely on antibody-based detection using chemiluminescent labels, as well as substrate-based chemiluminescent probes that emit light upon β-lactamase-mediated hydrolysis (often cephalosporin-based conjugates) that emit light upon enzymatic cleavage. These systems can detect β-lactamase activity with very high sensitivity, often exceeding that of fluorescence-based assays by several orders of magnitude [57,58,59].

Chemiluminescence is also used to monitor reactive oxygen species (ROS), particularly those generated by activated phagocytes during bacterial infection. Such ROS-responsive probes have been used to assess oxidative bursts, evaluate immune responses, and in some cases indirectly monitor antibiotic efficacy [60,61]. The main strengths of chemiluminescent systems are their high sensitivity and minimal background signal. However, they typically require careful control of reaction conditions, reagent stability, and timing to achieve reproducible, quantitative results in complex biological matrices [44,54,58,60].

### 4.4. Fluorescence

Fluorescence refers to light emission that occurs after a molecule absorbs photons. Depending on the electronic transition pathways, the emitted light can appear almost immediately. Fluorescence-based methods are predominantly used in the study of biological systems, mainly because they have a short lifetime, relatively high quantum yield, and are compatible with standard excitation sources used in microscopy and plate-based assays [28]. Fluorescent dyes and proteins are routinely used to assess bacterial physiology, viability, and membrane integrity. A common example is the SYTO 9/propidium iodide live/dead kit, which differentiates intact, viable cells from membrane-compromised ones following antibiotic exposure [62,63]. Genetically encoded fluorescent proteins, such as GFP, RFP, and mCherry, are widely applied in reporter strains to track stress responses, antibiotic-induced gene expression, quorum-sensing activity, or biofilm dynamics [46,64,65].

For AST, fluorescence underpins techniques such as high-content fluorescence imaging, flow cytometry, and microplate assays that monitor bacterial population changes in real time. Time-lapse fluorescence microscopy provides additional spatial and temporal resolution, enabling visualization of resistance development at the single-cell level or within structured communities like biofilms [46,66,67,68]. Despite these strengths, fluorescence depends on external excitation light, which introduces challenges such as photobleaching, signal decay, and sample autofluorescence, problems that can complicate measurements in complex biological matrices [28,46,64]. Recent advances have expanded fluorescence into more selective diagnostic platforms. Fluorescent nanosensors, quantum dots, and carbon-based fluorogenic probes have been engineered to activate only in the presence of specific antibiotic residues or resistance enzymes, improving selectivity and enabling multiplexed detection formats [69,70,71].

### 4.5. Comparison of Luminescent Techniques

Each luminescent modality offers distinct advantages depending on sensitivity needs, assay format, and compatibility with live-cell analysis. Bioluminescence and chemiluminescence offer exceptionally low background noise and high sensitivity, while fluorescence provides superior spatial resolution and is well suited for imaging-based assays. Table 2 summarises the key differences among the three modalities in the context of microbiology and antibiotic resistance studies.

## 5. Overview of the Luminescence Methods for β-Lactamases Detection

### 5.1. Bio-And Chemiluminescence

Luminescence-based strategies for detecting β-lactamases have accelerated noticeably in the past decade, largely because traditional phenotypic assays, culture growth, colour change, or prolonged incubation can no longer meet the demand for rapid diagnostics. A comparative overview of bioluminescent, chemiluminescent, and fluorescence-based assays developed for phenotypic β-lactamase detection is provided in Table 3. Bioluminescence and chemiluminescence offer something fundamentally different: a direct readout of enzymatic activity, extremely low LODs, and the ability to work with minimal amounts of biological material. In practice, this often translates into results obtained within minutes rather than hours or days. Recent studies addressed several complementary directions, from caged luciferins and bioluminescence resonance energy transfer (BRET) quenched bioluminescent probes to dioxetane-based chemiexcitation, relay-response amplification systems and even exploiting unusual β-lactam degradation metabolites such as hydrogen sulphide. Although these approaches differ chemically, they share a common goal: detecting β-lactamase activity in real time instead of relying on lengthy tests based on phenotypic resistance detection. Importantly, many of these probes have already been validated not only with purified enzymes but also with clinical isolates and, in some cases, complex matrices such as synthetic urine. The following section summarises the key advances of the last ten years, highlighting the sensitivity, specificity, workflow characteristics, and practical limitations of each method. All of these studies illustrate how luminescent technologies are reshaping phenotypic resistance detection toward faster and more sensitive assays.

Dai and co-authors [52] report the development of D-Bluco, an ultrasensitive bioluminogenic probe designed for rapid phenotypic detection of β-lactamase activity in viable bacteria. The probe is a cephalosporin-caged D-luciferin further conjugated to a DABCYL (4-(4-dimethylaminophenylazo)benzoic acid) quencher, which suppresses the substantial pre-activation background seen with earlier caged luciferins. Upon β-lactam-ring hydrolysis, free D-luciferin is released and produces light in a luciferase/ATP reaction. Mechanistic experiments show that conventional caged luciferins (Bluco, Am-Bluco) are partially oxidized by luciferase itself, generating background bioluminescence, whereas D-Bluco’s DABCYL moiety eliminates this via BRET. D-Bluco responds to multiple clinically relevant β-lactamases, including TEM-1, KPC-3, IMP-1, BlaC, AmpC, and OXA-48, with LODs of 0.01–0.1 femtomoles and catalytic efficiencies (kcat/Km) up to 5 × 10^6^ M^−1^s^−1^. In engineered *E. coli* overexpressing TEM-1, the probe detects as few as 10^2^ cfu/mL after 1 h. Clinical isolates (*E. coli*, *Klebsiella pneumoniae* and *Enterobacter cloacae*) express lower enzyme levels, so the authors developed a simplified RAPID-BLI workflow: 15 min lysis with 0.5% CHAPS, 15 min incubation with D-Bluco, followed by immediate luminescence readout. Using this protocol, β-lactamase-positive clinical isolates spiked into synthetic urine were detected at 10^2^–10^3^ cfu/mL within 30 min, well below the CDC diagnostic threshold for UTIs—urinary tract infections (≥10^5^ cfu/mL). Signal suppression by avibactam and clavulanic acid confirmed the specificity of the β-lactamase. All experiments were performed in vitro/ex vivo using bacterial cultures and clinical isolates; no human or animal studies were conducted. The research demonstrates that combining enzymatic caging with BRET-based quenching is a generalisable strategy for eliminating background in bioluminogenic probes, and positions D-Bluco as one of the fastest and most sensitive phenotypic assays for detecting β-lactamase-mediated resistance directly in urine-like samples.

The study of Van Almsick et al. [53] introduces a bioluminescence-based carbapenem susceptibility detection assay (BCDA) enabling rapid phenotypic identification of CRE (both CP and non-CP) and CP-CRA (*A. baumannii)* within 2.5 h from culture. The method measures bacterial viability through ATP-dependent luciferase bioluminescence (BacTiter-Glo) after short incubation with or without meropenem. Colonies grown on Columbia blood agar or MacConkey agar were suspended in MH/AST broth, split into a growth control (G) and an antibiotic tube (A), and incubated at 37 °C for 2.5 h with shaking; CRE were tested with 1 mg/L meropenem (liquid), while CRA required 10 mg/L (disk-derived). After incubation, lysed samples were mixed 1:1 with BacTiter-Glo reagent, and light emission was recorded, with results normalized as relative induction (ri). The study included 110 CRE and 65 CRA isolates: 106 carbapenemase producers (KPC; NDM, VIM, IMP and OXA), 22 non-CP-CRE (e.g., AmpC overexpression plus porin loss), and 47 meropenem-susceptible controls. All resistant strains were also phenotypically (determination of MICs) and genotypically confirmed by automatic and PCR detection, respectively. Using 10 mg/L meropenem and a cutoff of 36% ri, the BCDA detected CP-CRA with 100% sensitivity and specificity. For CRE (excluding *Citrobacter freundii*), the optimized 1 mg/L protocol and a 23% ri cut off yielded 98% sensitivity and 96% specificity: one OXA-48 *K. pneumoniae* with low minimal inhibitory concentration, MIC (0.25 mg/L) and one susceptible *E. coli* were misclassified. Non-CP-CRE were correctly identified except *C. freundii*, which showed no induction and could not be distinguished from susceptible *C. freundii*. A transition zone of 19–32% ri caused uncertainty for some OXA-type producers, consistent with known phenotypic detection difficulties. The assay performed entirely in vitro on cultured isolates (no patient samples), but the authors note potential applicability to other matrices such as blood cultures. Overall, 172/177 Gram-negative bacteria were correctly classified, corresponding to ~99% sensitivity and 98% specificity, offering a rapid and inexpensive phenotypic tool capable of detecting both carbapenemase-mediated and non-carbapenemase resistance, with limitations for low-MIC OXA-type producers and *C. freundii*.

Maity and co-authors [59] described the development of a cephalosporin–chemiluminescent conjugate (CCP), a dioxetane-based β-lactamase probe that enables chemiluminescent detection of β-lactamase activity with a sensitivity four orders of magnitude higher than the fluorescent substrate fluorocillin. CCP links a cephalosporin to a dioxetane via a thioether bond; β-lactamase hydrolysis releases a thiophenol-triggered dioxetane that undergoes 1,4-elimination to produce a phenolate intermediate, which decomposes to emit light. Unlike classical phenolic dioxetanes requiring pH~10, the thiophenol leaving group (low pKa) allows efficient chemiluminescence at neutral pH and also enables synthesis under mild basic conditions. The probe was synthesised through a 10-step convergent route involving allyl-protected cephalosporin chloride and a thiophenol–adamantane vinyl ether precursor, with dioxetane formation via singlet-oxygen oxidation and final Pd-catalysed deprotection. CCP was tested with several β-lactamases (TEM-1, CTX-M-15, NDM-1, OXA-1; all at concentrations of 1 nM), showing universal substrate behaviour and a 20-fold chemiluminescence increase over background within 30–60 min at room temperature, with no signal in PBS. Sensitivity assays using TEM-1 demonstrated a LOD of 200 fM, whereas fluorocillin detected only ≥5 nM, confirming the ~10,000-fold performance improvement. The probe distinguished β-lactamase-positive *E. coli* lysates (10^6^ CFU/mL) from negative controls with ~20-fold signal enhancement. Bacterial titration showed CCP could detect as few as 10^5^ CFU/mL, matching typical bacterial loads in UTIs and outperforming fluorocillin (limit ~10^7^ CFU/mL). CCP was further validated with clinical *E. coli* UTI isolates (5 × 10^5^ CFU/mL), correctly identifying 5/6 ampicillin-resistant strains and 5/6 susceptible strains using a chemiluminescence cutoff derived from susceptible controls. All experiments were conducted in vitro using purified enzymes, bacterial lysates, and clinical isolates; no in vivo or patient-sample fluid testing was performed. Overall, CCP provides the first chemiluminescent cephalosporin probe for β-lactamase and dramatically improves analytical sensitivity, showing promise for point-of-care (POC) diagnostics aimed at rapid phenotypic detection of β-lactamase-mediated resistance.

Gui et al. [72] report an electrogenerated chemiluminescence (ECL) biosensor for β-lactamase detection based on a ruthenium–ampicillin conjugate (Ru-Amp), where the Ru(phen)_2_(cpaphen)^2+^ fluorophore is covalently linked to ampicillin, enabling the probe to function simultaneously as the β-lactamase-specific recognition element and the ECL luminophore. Upon β-lactamase-catalysed hydrolysis of the β-lactam ring, Amp is converted into a secondary amine that strongly enhances Ru-based ECL emission, allowing direct readout of enzymatic activity. Ru-Amp was immobilized in high density on thiophenemalonic acid–reduced gold nanoparticles (TA@AuNPs) through electrostatic and π–π interactions, forming Ru-Amp/TA@AuNPs nanocomposites that were drop-cast onto a CNTs–Nafion–modified glassy carbon electrode (CNTs-Nf/GCE). The nanocomposites formed stable films with excellent ECL behavior, as verified by SEM, TEM, UV–vis spectroscopy, and cyclic voltammetry. The biosensor exhibited a quantitative response to β-lactamase from 50 pg/mL to 100 ng/mL in PBS (pH 7.4), with LOD of 37 pg/mL (S/N = 3) and a regression of I = 682.8 log c + 125 (R = 0.9911). The device showed high stability (RSD 0.89% over 14 potential cycles), good repeatability (RSD 1.75%, *n* = 5), acceptable one-week storage stability (<1.5% signal decrease), and excellent selectivity, with no significant ECL interference from alkaline phosphatase or thrombin at high concentrations. Control experiments confirmed that TA@AuNPs and Ru complexes did not inhibit β-lactamase activity. All experiments were performed in vitro using purified ampicillin (Amp) β-lactamase and controlled interference proteins; no bacteria, clinical isolates, or patient samples were tested. Overall, the Ru-Amp/TA@AuNPs ECL platform provides a simple, low-cost, and ultrasensitive strategy for detecting β-lactamase activity and demonstrates a generalizable approach for Ru-based ECL biosensing.

Gholap and co-workers [73] in their study identify hydrogen sulfide as a previously unrecognised biodegradation metabolite generated when β-lactamases hydrolyse sulfur-containing β-lactam antibiotics, and leverages this chemistry to create a chemiluminescent assay for detecting β-lactam resistance. Three dioxetane probes (1: disulfide-masked, 2: selenosulfide-masked, 3: dinitro-sulfonamide thiol probe; 25 μM) were tested in PBS pH 7.4 at 27 °C with cefalexin (1 mM) ± *Bacillus cereus* β-lactamase (βLBC, 10 U/mL). All probes responded to enzyme-driven biodegradation, but probe 1 produced the strongest turn-on (62-fold S/N after 2.5 h). Screening nine sulfur-containing β-lactams showed that only five (sulopenem, faropenem, cefalexin, ceftizoxime, cefazolin) generate strong H_2_S-dependent responses, while others fail due to alternative degradation pathways. Kinetic measurements over 2 h confirmed that all five produce high chemiluminescence (S/N 25–120) with probe 1 in the presence of βLBC and negligible background without enzyme. Using sulopenem, seven β-lactamases were compared: five (βLBC, KPC-1/2, NMCA, SPM-1, Bla-1) activated probe 1 strongly, whereas OXA-11 and VIM-15 did not. RP-HPLC during ceftizoxime (2 mM) hydrolysis revealed formation of the expected disulfide-cleavage byproduct A only when βLBC was present, directly confirming H_2_S generation; similar results were obtained for cefalexin. Probe 1 was ~10× more sensitive than Ellman’s reagent for detecting enzymatically generated H_2_S. In functional bacterial assays, resistant strains (OD600 ≈ 1.0) incubated with sulopenem or ceftizoxime (1 mM, 30 min) followed by probe 1 produced high S/B values (7–14 for sulopenem, 18–245 for ceftizoxime) compared with sterile controls. In a one-step 15-min assay with cefazolin (1 mM) and probe 1, resistant *K. pneumoniae* (KPC-2) and *E. coli* (CMY-2) showed strong light emission (S/B 30 and 41), while susceptible reference strains showed only background; inhibition with 3-aminophenylboronic acid reduced signal by 80–86%, verifying β-lactamase dependence. All experiments were in vitro with purified enzymes or cultured bacteria; no patient samples were tested. The work establishes H_2_S as a mechanistic β-lactam degradation marker and demonstrates a sensitive chemiluminescent platform for detecting β-lactamase-mediated antibiotic resistance.

Das et al. [74] in their study report the first chemiluminescent carbapenem-based molecular probe (CPCL) designed to detect carbapenemase activity directly in live bacteria. CPCL consists of a carbapenem core linked via a 4-hydroxymethylphenyl self-immolative spacer to a phenoxy-dioxetane luminophore; enzymatic hydrolysis of the β-lactam ring triggers rapid 1,8-elimination, generating a phenolate dioxetane that chemiexcites to emit green light. CPCL (typically 10 μM in PBS pH 7.4 with 5–10% DMSO) was evaluated against diverse β-lactamases. The metallo-carbapenemase SPM-1 (1 U/mL) produced an immediate and intense chemiluminescence burst peaking within ~5 min (55-fold over control), whereas the non-carbapenemase Bla-1 yielded only background-level signal. CPCL also detected clinically important enzymes including VIM-15, KPC-1, and NMCA, and the LOD for SPM-1 was 0.06 mU/mL. RP-HPLC analysis of CPCL (100 μM) incubated with SPM-1 confirmed formation of the expected phenolate intermediate and the chemiexcited benzoate species, while controls showed negligible hydrolysis. A fluorescent analogue (CPFL) based on umbelliferone failed to respond due to its higher pKa, underscoring the sensitivity advantage of chemiluminescence. The probe was then applied to bacterial cultures: imipenem-resistant *Pseudomonas aeruginosa* (IMP-2) and *K. pneumoniae* (KPC-2) produced strong chemiluminescence signals when whole colonies (≈5 × 10^8^ CFU/mL) or broth suspensions were mixed with CPCL, while imipenem-susceptible *E. coli* and sterile controls generated minimal light. The assay detected carbapenemase activity down to ~10^7^ CFU/mL and could be inhibited selectively using EDTA (IMP-2) or 3-aminophenylboronic acid (KPC-2), confirming mechanism specificity. All measurements were performed in vitro with purified enzymes and cultured bacterial isolates; no human samples were tested. Overall, CPCL enables rapid (<10 min), sensitive, phenotypic detection of carbapenemases and carbapenemase-producing organisms (CPOs), offering potential for fast diagnostic testing complementary to PCR-based genotyping.

The study of Shelef and co-authors [75] aimed to develop a fast, general method for bacterial identification and characterization based on enzymatic activity fingerprints measured with an ultrasensitive chemiluminescent probe array rather than growth, PCR, or MS-based typing. The authors designed a 12-probe panel built on ortho-acrylate-substituted phenoxy-adamantyl-1,2-dioxetane luminophores, each masked with a different enzyme-specific trigger (β-glucuronidase, β-galactosidase, phosphatase, leucine aminopeptidase, pyroglutamyl-peptidase, nitroreductase, NQO1, β-lactamase, penicillin-G amidase, N-acetyl hydrolase, and a vicinal-diol oxidative-cleavage control). Upon enzymatic cleavage of the triggering group, a self-immolative linker releases the phenolate dioxetane, which undergoes chemiexcitation to give a bright green CL signal in aqueous buffer. First, a phosphatase probe was compared to fluorescent (umbelliferone) and colorimetric (p-nitrophenol) analogues, showing ∼25–125-fold lower LODs and much higher S/N for recombinant alkaline phosphatase and *Staphylococcus aureus* suspensions in PBS pH 7.4 at 37 °C. The full array (typically 10 μM probe, PBS 100 mM, pH 7.4, 0.1% DMSO, 37 °C, 60 min) was then applied to intact cells from 29 strains of clinically relevant bacteria (16 Gram-positive and 13 Gram-negative), recording total light emission over time to generate 12-dimensional “fingerprints”. Principal component analysis and 1-nearest-neighbor classification allowed 100% correct strain-level assignment after 60 min, while a reduced set of six probes still achieved 89.6% accuracy. Distinct signatures included high β-glucuronidase activity in *E. coli*, strong phosphatase activity in *Streptococcus pyogenes*, and characteristic pyroglutamyl-peptidase/leucine aminopeptidase patterns in *P. aeruginosa*; the β-lactamase and penicillin-G amidase probes also report on β-lactam-related resistance enzymes. The method correctly grouped unknown strains by species based on χ^2^ similarity to the database and produced interpretable mixed fingerprints for *E. coli*/*S. aureus* co-cultures, but unknown species not present in the database could only be assigned by nearest functional similarity, and some probes show modest background hydrolysis. Overall, this chemiluminescent array provides a rapid (~90 min), highly sensitive in vitro platform for functional classification of bacterial pathogens and enzymatic resistance phenotypes, with potential for future diagnostic and therapeutic-guiding applications.

The main goal of the study of Yang et al. [76] was to develop a simple chemiluminescent assay for rapid discrimination of β-lactam-resistant bacteria (BLRB) by exploiting hydrogen sulfide as a β-lactam biodegradation metabolite. The authors designed HS-CL, a “turn-on” phenoxy-dioxetane probe bearing a 2-iodobenzoyl ester as an H_2_S-reactive trigger; nucleophilic attack by HS^−^ cleaves the ester, liberates a phenolate-dioxetane, and initiates CIEEL (chemically initiated electron exchange luminescence) chemiexcitation, producing a green-emitting benzoate. In buffer and saline (40 μM HS-CL, 0.1–1% DMSO), Na_2_S produced an ∼18-fold chemiluminescence increase with a linear range of 5–100 μM (R^2^ = 0.9822) and an LOD of 1.02 μM, while the probe showed high selectivity over common cations, anions, ROS, and biothiols. At the enzymatic level, β-lactamase (10 U/mL) plus β-lactams (1 mM cefalexin, ceftizoxime, or cefazolin) generated H_2_S that activated HS-CL, with ceftizoxime giving the strongest response; HS-CL was more sensitive than Ellman’s reagent under identical conditions. For bacterial tests, commercial strains (10^4^ CFU/mL in LB) were preincubated with ceftizoxime (1 mM, 37 °C, 3 h) and then probed with HS-CL (40 μM), giving strong chemiluminescence only for β-lactam-resistant strains *A. baumannii* ATCC 19606, methicillin-resistant *S. aureus* (MRSA), *E. coli* ATCC 35218, and *K. pneumoniae* ATCC 700603, but not for susceptible *S. aureus* ATCC 6538 or *E. coli* ATCC 25922; the bacterial LOD was 10^2^–10^3^ CFU/mL, with highly significant signal differences (*p* < 0.0001). Finally, in six clinical isolates (*E. coli*, *K. pneumoniae* and *P. aeruginosa*), HS-CL correctly differentiated β-lactam-resistant from susceptible strains in a double-blind study, in agreement with MIC-based classification. The work demonstrates an in vitro/ex vivo chemiluminescent H_2_S readout for phenotypic β-lactam resistance that is relatively fast and equipment-light, though it depends on sufficient H_2_S release (thus on antibiotic structure and β-lactamase pathway), requires a few-hour antibiotic incubation, and has so far been validated only with ceftizoxime and a limited strain set.

Ma with co-authors [77] study introduces a relay-response chemiluminescence assay for rapid detection of bacterial resistance to β-lactam antibiotics by coupling a thiophenol-releasing β-lactam pro-substrate with a thiophenol-activated phenoxy-dioxetane reporter. The system uses dinitrophenylsulfonyl-caged chemiluminescent probe CL-1 (a 3-chloro, ortho-acrylate phenoxy-adamantyl-1,2-dioxetane) and three thiophenol-caged β-lactams: BL-1 (cephalothin analogue, first-generation cephalosporin), BL-2 (cefotaxime analogue, third-generation cephalosporin) and BL-3 (meropenem analogue, carbapenem). Upon hydrolysis of the β-lactam ring by β-lactamases, free thiophenol is released, which rapidly attacks the dinitrophenylsulfonyl group, decaging CL-1 and triggering dioxetane decomposition via a CIEEL mechanism with green chemiluminescence at ~509 nm. Assays were performed in PBS pH 7.4 at room temperature in 96-well plates with 10 μM CL-1 and typically 50 μM BL-n, using either purified enzymes (TEM-1, CTX-M-9, KPC-2, NDM-1, NDM-4, NDM-12, VIM-1, IMP-1, CphA, L1, AmpC, OXA-1) or β-lactamase-expressing bacteria (TEM-1-producing *E. coli*, CTX-M-9-producing *E. cloacae*, KPC-2-, NDM-1-, VIM-1-producing *K. pneumoniae*, AmpC-producing *E. cloacae*) plus β-lactamase-negative *E. coli* and *Bacillus subtilis* controls. CL-1 alone was stable toward amino acids, media (LB, MH) and β-lactamase-negative bacteria, but showed >10^3^–10^4^-fold signal increase with thiophenol; in the relay mode, the TEM-1 LOD was 0.026 nM using BL-1, while NDM-1 was detected down to 0.013 nM (CL-1/BL-2) and 0.0049 nM (CL-1/BL-3). By combining BL-1, BL-2 and BL-3, the assay discriminates narrow-spectrum β-lactamases (TEM-1, OXA-1; strong signal only with BL-1), ESBLs (CTX-M-9; signal with BL-1 and BL-2) and carbapenemases (KPC-2 and MBLs; strong signal with BL-1 and BL-2, BL-3 particularly for MBLs such as NDMs, VIM-1, IMP-1, CphA, L1), reflecting the hydrolysis scope toward first-and third-generation cephalosporins versus carbapenems. In bacterial suspensions (~10^7^ CFU/mL, 6 min readout) and in four clinical *K. pneumoniae* isolates characterized by MICs to cefazolin, ceftriaxone and imipenem, chemiluminescence patterns correlated with phenotypic resistance profiles, enabling fast ex vivo resistance typing without cell permeabilization. The method is highly sensitive and synthetically modular but currently limited to in vitro/ex vivo use, still relies on cultured bacteria, and the multistep relay may complicate real-time kinetic analysis or direct application to non-β-lactamase resistance mechanisms.

In summary, the reviewed studies demonstrate that both bioluminescence and chemiluminescence have evolved into powerful tools for detecting β-lactamase activity, moving far beyond early proof-of-concept experiments. Bioluminescent probes stand out for their exceptional sensitivity and rapid enzymatic response, while chemiluminescent systems offer outstanding signal-to-noise ratios, flexible probe design, and the ability to differentiate between β-lactamase classes through tailored activation mechanisms. What unites these approaches is their reliance on true enzymatic function, providing a more accurate picture of resistance than genetic detection alone. At the same time, none of these methods are currently suitable for routine clinical application. Challenges such as probe stability, differences in enzyme expression across isolates, the need for short antibiotic incubations, and variable matrix effects still influence their performance. Despite these hurdles, the progress is undeniable: several luminescent assays now detect 10^2^–10^3^ CFU/mL within 10–30 min—levels that would have been implausible for phenotypic testing only a few years ago. Collectively, these luminescent approaches are pushing β-lactam resistance diagnostics toward faster turnaround, higher sensitivity, and more functionally meaningful results, and are well-positioned to become practical components of future POC workflows.

### 5.2. Fluorescence

Fluorescent methods have become some of the most versatile tools for studying antibiotic resistance in living bacteria. Unlike purely molecular tests, fluorescence readouts can directly report on enzyme activity, cell viability, drug accumulation or metabolite production in real time and at either single-cell or population level. β-Lactamases are attractive targets for fluorescent probe design in the context of antibiotic resistance because they are abundant, mechanistically well understood, and closely associated with clinically relevant phenotypes. In recent years, a wide range of enzyme-activatable probes has been reported, encompassing everything from simple “off–on” substrates for broad-spectrum β-lactamase screening to highly engineered, self-immobilizing and near-infrared (NIR) probes suitable for in vivo imaging or point-of-care testing (POCT). In the following subsections, we summarise these developments with a focus on β-lactamase-targeted systems, including broad-spectrum fluorogenic probes, tools selective for SBLs or MBLs, carbapenemase-directed sensors, and indirect or nanomaterial-based platforms, as well as an AI-assisted mobile system that couples fluorescence readout with automated data analysis.

**Figure 2:**
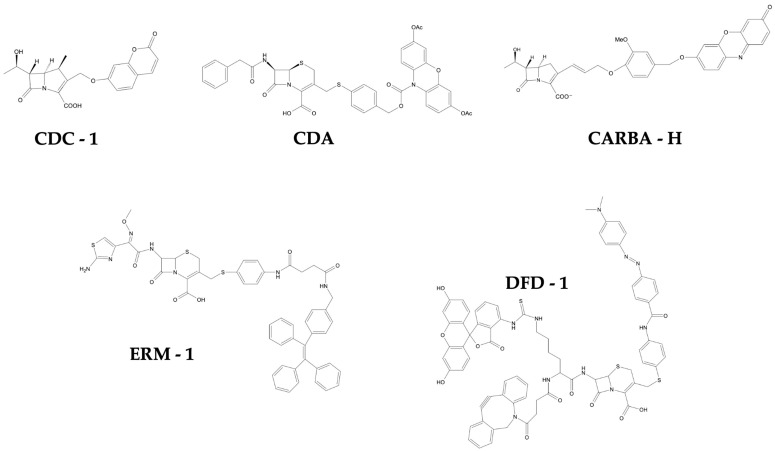
Chemical structures of selected luminescent probes discussed in the text.

Xie and colleagues [78] developed CDA (Cephalosporin Caged Diester Amplex Red analogue, Figure 2), a dual-caged fluorogenic resorufin-based probe for the detection of a broad spectrum of β-lactamases. The probe is based on a 3,7-diesterphenoxazine (Amplex Red analogue) core. It is caged by two acetate groups and a cephalosporin moiety, which functions as a β-lactamase substrate. Activation of the probe by a β-lactamase results in the release of the intermediate that is subsequently hydrolysed by bacterial esterases and oxidised to form a fluorescent resorufin. The minimal CDA amount required to detect β-lactamase/esterase activity was determined to be 100 nM, while 10 μM produced optimal signal strength (λ_ex/em = 570/585 nm). Even in the absence of H_2_O_2_, incubation of CDA with TEM-1 and an esterase resulted in a 1200-fold increase in fluorescence intensity, likely caused by the presence of dissolved oxygen in the solution. The capability of CDA to detect β-lactamases beyond TEM-1 was assessed using a panel of clinically prevalent β-lactamases, including the *Mycobacterium tuberculosis* specific BlaC, cephalosporinases AmpC and the carbapenemases KPC-3, IMP-1 and OXA-48. CDA successfully detected all β-lactamases at concentrations as low as 1 femtomole after 2 h of incubation at room temperature. Subsequently, the probe’s ability to detect TEM-1-producing *E. coli* in the absence of additional oxidizing agents was evaluated. Detectable fluorescence was observed only at high bacterial concentrations (10^7^ CFU mL^−1^). To determine whether higher concentrations of H_2_O_2_ could permeate bacterial membranes and promote the oxidation of reduced resorufin, serial dilutions of H_2_O_2_ were incubated with CDA and varying concentrations of TEM-1-producing and β-lactamase–negative *E. coli*. Using 1 mM of CDA/H_2_O_2_ facilitated the detection of 10^5^ CFU mL^−1^ of TEM-1-producing *E. coli* within 2–3 h, while detection of 10^4^ CFU mL^−1^ required 4 h. A 20-h preincubation with CDA further enhanced the sensitivity, allowing detection of as few as 10^3^ CFU mL^−1^. In addition, the assay successfully detected 10^5^ CFU mL^−1^ of IMP-1-producing *E. coli* and 10^5^–10^6^ CFU mL^−1^ of KPC-3-producing *E. coli* within 2 h. Moreover, to investigate the ability of CDA to detect *K. pneumoniae*, two clinical isolates (KPC-positive and KPC-negative) were incubated with CDA and H_2_O_2_. Similarly to KPC-3-producing *E. coli*, 10^6^ CFU mL^−1^ of KPC-positive *K. pneumoniae* was detected within 2–3 h. These results indicate that the CDA/H_2_O_2_ assay is capable of detecting clinical strains that produce cephalosporinases AmpC and carbapenemases. Furthermore, the CDA/H_2_O_2_ assay was assessed for its effectiveness in screening urine samples. Synthetic urine samples (50 mL) were inoculated with β-lactamase–positive and β-lactamase–negative strains of *E. coli*, *K. pneumoniae*, *E. cloacae* and *Serratia marcescens* at 10^3^, 10^4^, or 10^5^ CFU mL^−1^. Following two-step filtration, the CDA/H_2_O_2_ assay achieved a LOD of 10^3^ CFU mL^−1^ for all tested β-lactamase-producing bacteria (BLPB) within 2 h, whereas 10^5^ CFU mL^−1^ could be detected within 1 h. Collectively, these findings demonstrate that the CDA/H_2_O_2_ assay is an effective tool for reporting UTIs. Moreover, the incorporation of a dual caging mechanism results in lower background fluorescence and higher detection sensitivity compared to single caged fluorogenic probes. This property makes CDA a promising tool for the initial screening for broad-spectrum β-lactamase activity, and for the diagnosis of ESBL-producing and carbapenem-resistant pathogens.

Chen et al. [79] designed CDG-1, an off-on fluorescein-based fluorogenic probe for the detection of a broad spectrum of β-lactamases. CDG-1 consists of a cephalosporin moiety, a Tokyo Green fluorophore (a fluorescein derivative) and a benzyl ether linker for increased stability of the probe. CDG-1 can be utilised on its own or in conjunction with the fluorescent immuno-magnetic separation (FIMS) technique. The integration of β-lactam-antibody-bearing magnetic beads facilitates the detection of specific β-lactamases and improves the sensor’s sensitivity. Enzymatic cleavage of the probe’s β-lactam ring releases the fluorescent Tokyo Green from its bond with the benzyl ether linker. This is followed by a 10-fold increase in fluorescence intensity after 15 min of incubation (λ_ex/em = 490/525 nm). The performance of the CDG-1 probe was compared with a TEM-1-specific commercial ELISA kit using milk samples spiked with TEM-1 and cephalosporinase. Although both the ELISA kit and CDG-1 probe had a LOD of 10^−3^ U mL^−1^ for TEM-1, the ELISA assay required 4 h and detected only TEM-1, whereas CDG-1 detected both β-lactamases within 15 min. This demonstrates its rapid response and broad-spectrum detection capabilities. The effectiveness of the FIMS sensor was subsequently evaluated. Two types of magnetic beads were added to the milk samples, each conjugated to a specific antibody (either anti-TEM-1 or anti-cephalosporinase). The commercial ELISA kit detected TEM-1 at ≥10^−2^ U mL^−1^ in 4 h, whereas the FIMS sensor achieved a LOD of 10^−5^ U mL^−1^ within 30 min. Furthermore, the FIMS sensor detected 93.3% of the positive samples, in comparison to the ELISA kit’s detection rate of 33%, demonstrating its superior sensitivity and detection efficiency. To improve its suitability for POCT, the assay can be simplified by using a handheld UV lamp as the excitation source and a smartphone as the detector, achieving a LOD of 10^−2^ U mL^−1^ for TEM-1. The FIMS sensor exhibits considerable potential for rapid, POC screening for β-lactamases in milk and clinical samples. In comparison with ELISA kits, it is faster, more sensitive, has a broader detection range, and requires no sample pretreatment.

Guo et al. [80] introduced CyPA-L, a dual-modal probe that facilitates rapid detection of β-lactamases and BLRB. The probe combines a NIR cyanine fluorophore (Cy) with a cephalosporin intermediate which functions as an enzyme-cleavable masking group. In the probe’s intact form, the π-conjugation of the cyanine dye is disrupted, resulting in quenched fluorescence and a negligible photoacoustic signal. When the β-lactam ring is hydrolysed by a β-lactamase, the π-conjugation is restored, leading to a significant increase in both NIR fluorescence intensity and photoacoustic emission. Owing to its PA property, this approach exhibits a higher signal-to-noise ratio than conventional fluorescent probes. The optimal conditions for detecting TEM-1 with CyPA-L were reported to be 37 °C, at pH 7.4. Marked increases in fluorescence intensity were observed when the CyPA-L probe was incubated with TEM-1 and with a lysate derived from β-lactam-resistant *E. coli*. Incubation of CyPA-L with β-lactam-resistant *E. coli* lysate pretreated with potassium clavulanate (β-lactamase inhibitor) resulted in no increase in fluorescence intensity. This confirms that the probe’s activation was due to β-lactamase-mediated hydrolysis. The LOD of the probe was determined to be 2.1 nM for TEM-1 activity and 3.1 × 10^5^ CFU mL^−1^ for the detection of BLRB (λ_ex/em = 725/763 nm). The selectivity of CyPA-L for BLRB was evaluated using a panel of bacterial strains comprising *Enterococcus faecalis*, *S. aureus* including MRSA, *P. aeruginosa*, *K. pneumoniae*, *E. coli* and β-lactam-resistant *E. coli*. A substantial increase in fluorescence intensity was observed exclusively upon incubation with the β-lactam-resistant *E. coli* lysate. Subsequently, the potential interference of multidrug-resistant (MDR) bacteria in BLRB detection by the CyPA-L probe was investigated. Incubation with a lysate of a MDR *P. aeruginosa* clinical strain resulted in a significant increase in fluorescence intensity and PA signal activation. No fluorescence or PA signal was observed after pretreatment with potassium clavulanate. These findings indicate that the CyPA-L probe is a robust tool for the selective detection of BLRB. CyPA-L exhibits high selectivity and a rapid response time for the detection of TEM-1, requiring only seven minutes for complete activation of its NIR fluorescence and PA signals. Furthermore, it facilitates precise identification of antibiotic-resistant bacteria within 40 min. In combination with a portable PA device, CyPA-L offers a promising platform for POCT.

**Figure 3:**
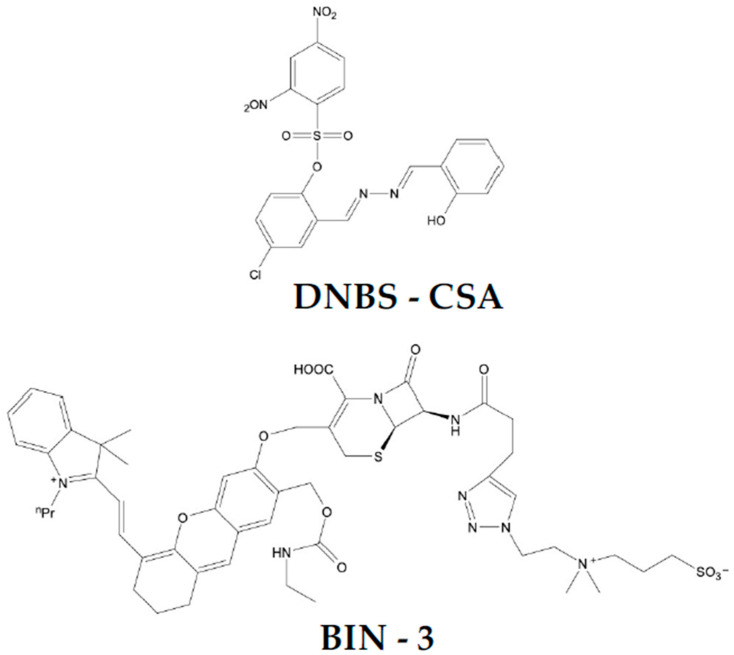
Chemical structures of selected luminescent probes discussed in the text.

Chen et al. [81] developed BIN-3 (Figure 3), a hydrophilicity-switching, self-immobilizing, NIR fluorogenic probe, designed for highly sensitive in vivo imaging of infections caused by BLPB. It consists of a cephalosporin core conjugated to a NIR fluorescent dye (HD), onto which a carbamate leaving group is attached. An additional zwitterionic, hydrophilic moiety improves aqueous solubility and minimises non-specific uptake prior to activation. Upon β-lactam ring cleavage, the probe loses its hydrophilic elements, forming a reactive quinone methide intermediate. Its highly hydrophobic nature promotes its accumulation within BLPB and covalent binding to bacterial proteins (nucleophiles). Incubation of BIN-3 with TEM-1 produced a robust NIR fluorescence signal (λ_ex/em = 660/710 nm). No change in fluorescence intensity was observed when TEM-1 was co-incubated with avibactam, a β-lactamase inhibitor. The probe’s selectivity was further confirmed using a panel of biologically relevant analytes, including catalytic proteins (lysozyme, β-glucosidase and lipase), a non-catalytic protein (bovine serum albumin, BSA) and small molecules (glutathione, lysine, cysteine and tryptophan). To further investigate the selectivity of BIN-3, the probe was incubated with lysates from β-lactamase–producing *E. cloacae* and β-lactamase–negative *E. coli*. A significant increase in fluorescence intensity was observed exclusively in the β-lactamase–producing strain. This signal was markedly reduced by co-incubation with avibactam. Additionally, the ability to selectively label individual BLPB in the presence of β-lactamase–negative strains was investigated by incubating BIN-3 with a mixture of β-lactamase-producing *E. cloacae* and β-lactamase–negative *E. coli*. Only β-lactamase–positive *E. cloacae* were selectively labeled. The concentration of HD fluorophores within β-lactamase–positive *E. cloacae* was determined to be over 1500 times higher (269.59 μM) than in the incubation medium (0.164 μM). The probe’s performance in a mouse infection model was subsequently evaluated (intramuscular injection with 5 × 10^8^ CFU of β-lactamase–producing *E. coli*). BIN-3 produced a distinct fluorescence signal at the infection site within one hour. The signal gradually intensified over the following hours, reaching a signal-to-background ratio (SBR) of over 10 after 3 h. After 10 h, a comparable fluorescence signal remained detectable at the infection site, with an SBR of ~9. The in vivo sensitivity of BIN-3 was further assessed in mice infected with TEM-1-producing *E. coli*. Whole-body fluorescence imaging revealed a strong correlation between the fluorescence intensity at infection sites and the quantity of bacteria present. The LOD for BLPB was determined to be 2 × 10^6^ CFU, with a SBR of 3.0. Moreover, the BIN-3 probe’s capability to distinguish BLPB from susceptible bacteria in living mice was tested using β-lactamase–negative *E. coli* and TEM-1-producing *E. coli*. A strong fluorescence signal was observed exclusively at the infection site of TEM-1-producing *E. coli*, persisting for over 10 h. In addition, the potential of BIN-3 for in vivo assessment of β-lactamase inhibitor efficacy was tested in mice intramuscularly inoculated with TEM-1- and NDM-1-producing *E. coli* strains. Subsequent to this, the mice were intravenously administered either avibactam or ebselen, followed by a BIN-3 injection 5 h later. Whole-body imaging demonstrated that fluorescence was suppressed only in mice infected with TEM-1-producing *E. coli*, possibly due to poor water solubility of ebselen. Collectively, these results indicate that BIN-3 could serve as a reliable tool for wash-free visualization of β-lactam-resistant bacteria and real-time in vivo monitoring of bacterial infections. Furthermore, BIN-3 could aid in the development of novel therapeutic agents and the optimization of drug dosing regimens.

Mao et al. [82] synthesised CFC-2, a coumarin-based self-immobilizing fluorogenic probe for the detection of BLPB. CFC-2 consists of a cephalosporin intermediate that functions as a β-lactamase recognition moiety and a difluoromethyl-substituted coumarin that acts as an activatable fluorophore. Upon enzymatic activation by a β-lactamase, CFC-2 generates a quinone methide (VI) intermediate, which serves as a highly reactive Michael acceptor for the covalent labelling of nearby bacterial proteins (potential nucleophiles). It was determined that the use of a higher concentration of the CFC-2 probe does not always result in an increased fluorescence turn-on ratio, presumably due to insufficient nucleophile availability. Enzymatic activation of 10 μM of CFC-2 with 0.5 nm of TEM-1 produced extensive fluorescence in the presence of 1 × 10^7^ CFU mL^−1^ β-lactamase-negative *E. coli*. Excitation at 360 nm resulted in a robust fluorescence signal when CFC-2 was incubated with 6.8 μM of TEM-1 at 37 °C for 2 h. Incubation of CFC-2 with 6.8 μM of BSA produced a negligible signal, demonstrating its specificity. Following 90-min incubation with TEM-1-producing *E. coli* at 37 °C and subsequent washing, a robust fluorescence signal was observed (λ_ex/em = 365/460 nm). Fluorescence microscopy was employed to further investigate the probe’s ability to label individual live bacterial cells with CFC-2. Following incubation with CFC-2 and subsequent washes, the β-lactamase-negative *E. coli* strain was nearly non-fluorescent. TEM-1-producing *E. coli*, MDR *A. baumannii* (ATCC BAA1605) and VIM-1-producing *K. pneumoniae* (NCTC 13440) demonstrated a significant increase in fluorescence intensity. NDM-1-producing *K. pneumoniae* (ATCC BAA2146) exhibited weaker fluorescence signal, but still substantially higher than that of the Βla-negative *E. coli*. These findings indicate that the CFC-2 probe can both detect and covalently label a broad range of BLPB. Despite the probe’s blue fluorescence, the impact of background autofluorescence is mitigated by its self-immobilizing property, which prevents fluorophore diffusion. 

Yu and colleagues [83] developed CDC-559, a coumarin-based fluorescent probe for the selective and specific detection of AmpC β-lactamase activity. CDC-559 consists of a 7-diethylaminocoumarin fluorophore and a 3-cephem-4-carboxylate (cephalosporin derivative) which serves as a β-lactamase substrate. The two moieties were chemically connected by a carbon–carbon double bond. The incubation of 10 μM of CDC-559 with 100 U mL^−1^ of AmpC for 10 min resulted in significant fluorescence quenching (turn-off response) of up to 93% (λ_ex/em = 440/539 nm), which was attributed to the formation of a conjugated imine moiety. In contrast, incubation with 100 U mL^−1^ of TEM-1 had a negligible effect on fluorescence intensity. The higher selectivity for AmpC over TEM-1 is likely due to the short linker at the 3′ position of the cephalosporin, which sterically hinders the active site of TEM-1. Following 30 min of incubation at 37 °C, the LOD for AmpC was determined to be 0.377 U mL^−1^ using the 3σ/κ method. CDC-559 was then used to assess the potency of known β-lactamase inhibitors, yielding IC_50_ values of 0.279 μM for sulbactam and 0.053 μM for tazobactam. Subsequently, the probe’s ability to distinguish between two cephalosporin-resistant strains (*S. aureus* ATCC 43300 and *E. cloacae* ATCC 13047) and two antibiotic-susceptible strains (*S. aureus* ATCC 25923 and *S. aureus* ATCC 29213) was investigated. It was determined that the fluorescence intensity of CDC-559 was barely affected by the antibiotic-susceptible strains. Although *S. aureus* ATCC 43300 and *E. cloacae* ATCC 13047 are both cephalosporin-resistant, the extent of fluorescence quenching varied between the two strains. Within 10 min, *E. cloacae* ATCC 13047 caused a rapid and substantial decrease in fluorescence intensity of approximately 76%. *S. aureus* ATCC 43300 caused only a modest reduction. These results demonstrate the CDC-559 probe’s potential for rapid screening of AmpC β-lactamase inhibitors and detecting AmpC-producing bacteria.

Chan et al. [84] designed DFD-1 (Figure 2), an off-on fluorogenic probe designed for the selective detection of AmpC-producing bacteria. The probe is based on a cephalosporin scaffold functionalised with a fluorescein isothiocyanate (FITC) fluorophore and a DABCYL quencher. These two elements form a fluorescence resonance energy transfer (FRET) pair that efficiently quenches the fluorescence of the DFD-1 probe. The probe’s selectivity for AmpC over other β-lactamases was achieved by attaching a sterically demanding dibenzocyclooctyne (DBCO) group to the 7′-position of the cephalosporin. Enzymatic hydrolysis of the cephalosporin’s β-lactam ring by AmpC causes the release of DABCYL from the 3′-position, resulting in a ~67 fold increase in fluorescence intensity (λ_ex/em = 488/516 nm). To anchor the probe to the bacterial cell membrane, an azide-functionalised lipid conjugate was used. Covalent binding between the DBCO group of DFD-1 and the lipid-azide conjugate occurs via copper-free click chemistry (SPAAC). This approach minimises the diffusion of the fluorescent dye, facilitating real-time cell visualization. Tests involving different lipid chain lengths indicated that LA-12 (C12) yielded the strongest fluorescence signal with minimal disruption to bacterial cell function. The probe’s specificity was confirmed with an inhibitor assay involving aztreonam (AmpC inhibitor) and clavulanic acid (class A and D β-lactamase inhibitor). The DFD-1 probe was subsequently evaluated for its effectiveness in fluorescence imaging using live AmpC-producing strains (*E. cloacae* ATCC 13047 and *P. aeruginosa* PAO1 ATCC 15692), TEM-1-producing control strains (*E. faecium* ATCC 51559 and MRSA ATCC BAA44) and two antibiotic-susceptible strains used as negative controls (*P. putida* OUS82 and *S. aureus* ATCC 29213). Each strain was treated with LA-12 for 1 h at 37 °C, followed by washing and 30-min incubation with DFD-1. The samples were then analysed using confocal fluorescence microscopy. A strong fluorescence signal was only observed in AmpC-producing strains. Significantly weaker signals were detected in the TEM-1-producing strains and almost no fluorescence was observed in the antibiotic-susceptible strains. Subsequent incubation of AmpC-producing strains with aztreonam resulted in a substantial reduction in fluorescence intensity during bacterial imaging, while incubation with clavulanic acid had minimal impact on their fluorescence intensity. Furthermore, *P. aeruginosa* PAO1 was used to assess the feasibility of employing flow cytometry for the quantification of AmpC-producing bacteria labelled with DFD-1 and LA-12. Antibiotic-susceptible *S. aureus* (ATCC 29213) and TEM-1-producing *S. aureus* (ATCC BAA44) were used as negative controls. Fluorescence signals were measured at 525 nm. In comparison to the control strains, *P. aeruginosa* PAO1 exhibited a substantial increase in fluorescence intensity. Pre-treatment with aztreonam significantly reduced the intensity of this signal. These results demonstrate that DFD-1 is a reliable probe for the detection and quantification of AmpC β-lactamase, as well as for the selective imaging of live AmpC-producing bacteria. Moreover, lipid-mediated anchoring of the probe provides a promising strategy for the precise and reliable screening of bacterial resistance in clinical settings, as well as for the evaluation of antibacterial agents, both in vitro and in vivo.

Chen et al. [85] introduced RLB-2, a NIR fluorogenic probe for the labelling of all SBLs. It consists of relebactam, an SBL inhibitor, and P-Mero4, a merocyanine dye that serves as a fluorophore. The interaction of the probe with SBLs results in the formation of a covalent bond. This leads to a reduced background signal and improved detection sensitivity in comparison to conventional diffusion-prone hydrolysing probes. The subsequent fluorescence turn-on ratio was reported to be as high as 300-fold (λ_ex/em = 685/720 nm). To investigate the labelling profile of the probe, RLB-2 was incubated with a panel of β-lactamases, which included class A (TEM-1, KPC-2), class B (NDM-1, IMP-1, VIM-27, CphA), class C (AmpC) and class D (OXA-1). All tested SBLs (class A, C and D) exhibited robust NIR fluorescence signal upon labelling with RLB-2, as demonstrated by in-gel fluorescence imaging. Pre-incubating TEM-1 with avibactam (an SBL inhibitor) prevented the covalent binding of RLB-2 to TEM-1, resulting in a substantially decreased fluorescence signal. No significant change in fluorescence was observed for any of the tested MBLs or for BSA, confirming the specificity of RLB-2 to SBLs. The probe’s ability to fluorescently label live SBL-producing bacteria was verified by incubating AmpC-producing *E. cloacae* (ATCC BAA-1143) with RLB-2, followed by imaging with fluorescence microscopy. Negative controls included avibactam-pretreated *E. cloacae* BAA-1143 and β-lactamase-negative *E. coli*. Strong fluorescence signal was detected from the AmpC-producing *E. cloacae* strain, even in the absence of a washing step. Both control strains showed negligible fluorescence signals. These findings illustrate the potential of RLB-2 for the wash-free labelling of SBLs and visualization of live SBL-producing bacteria. The study also demonstrated relebactam’s potential as a highly selective ligand for targeting SBL-producing bacteria.

Aw et al. [86] designed ERM-1 (Figure 2), an enzyme-sensitive reporter molecule that is capable of selectively localizing AmpC-producing bacteria in biofilms. ERM-1 is based on a modified cephalosporin scaffold which incorporates a sterically encumbered methoxyimino moiety. This modification increases the probe’s selectivity for AmpC and provides resistance to class A β-lactamases. Additionally, a tetraphenylethylene (TPE) fluorophore was covalently bound to the 3’ position of the cephalosporin through a 4-aminothiophenol linker. Activation of the probe by AmpC results in the release of the linked TPE, whose aggregation-induced emission (AIE) properties enhance the produced fluorescence signal. Extensive testing with β-lactamases (AmpC and TEM-1) and aztreonam indicated that ERM-1 is highly specific toward AmpC. Following the enzymatic reaction of ERM-1 with AmpC, a ~120-fold increase in fluorescence intensity was observed (λ_ex/em = 350/478 nm). The reaction with TEM-1 resulted in a ~40 fold increase due to the probe’s partial hydrolysis. The applicability of ERM-1 for live cell imaging was assessed using AmpC-producing *E. cloacae* and TEM-1-producing *E. coli* BL-21. An antibiotic-susceptible *E. coli* DH5α strain (ATCC 53868) was used as the negative control. The strains were incubated with 20 μM of ERM-1 at 37 °C for 1 h before imaging with confocal fluorescence microscopy. A significant increase in fluorescence intensity was detected exclusively in AmpC-producing *E. cloacae*, while TEM-1-producing *E. coli* exhibited only a weak fluorescence signal. No fluorescence was observed in the *E. coli* DH5α control strain or in AmpC-producing *E. cloacae* pretreated with aztreonam (AmpC inhibitor). These results demonstrate the ERM-1 probe’s capacity to selectively detect AmpC activity and label resistant bacteria. Furthermore, the strains were used to assess the feasibility of quantifying the specific labelling of AmpC-producing bacteria with ERM-1 using flow cytometry. The AmpC-producing *E. cloacae* strain exhibited a ~10-fold increase in fluorescence intensity compared to the control *E. coli* DH5α strain, while the TEM-1-producing *E. coli* strain displayed only a ~3-fold increase. The ability of ERM-1 to selectively localise and monitor bacterial biofilm formation was evaluated using the same bacterial strains as previously described. Biofilms were cultured on coverslips in LB broth at 37 °C for 24 h, after which they were treated with 20 μM of ERM-1 for 1 h. Confocal fluorescence microscopy revealed a strong fluorescence signal in biofilms formed by AmpC-producing *E. cloacae*, while those formed by TEM-1-producing *E. coli* exhibited only a weak fluorescence signal. No fluorescence was detected in AmpC-producing *E. cloacae* biofilms which were pretreated with aztreonam or in biofilms formed by the *E. coli* DH5α strain. These results indicate that the ERM-1 probe can be effectively used for the fluorescent imaging of AmpC-producing bacteria in biofilms. Furthermore, its use could provide valuable insights into the process of biofilm formation by antibiotic-resistant bacteria, potentially aiding the treatment of biofilm-related infections.

Mao et al. [87] developed CPC-1, a highly selective fluorogenic probe designed for the specific detection of MBLs. It is based on a carbapenem scaffold that functions as a quencher and umbelliferone that acts as a fluorophore. Unlike cephalosporin-based probes, CPC-1 shows no response to SBLs (KPC-3 and OXA-48), while being rapidly activated by MBLs (such as VIM-27, NDM-1 and IMP-1). Upon hydrolysis by VIM-27, CPC-1 exhibits a >200-fold increase in fluorescence intensity (λ_ex/em = 365/454 nm). To evaluate the probe’s sensitivity, serial concentrations of MBLs were incubated with 10 μM of CPC-1 for 45 min. The fluorescence intensity was measured and plotted against MBL concentration, yielding LODs of 31 pM for IMP-1, 11 pM for VIM-27 and 3 pM for NDM-1. In addition, an inhibition assay was conducted to confirm that the observed change in fluorescence intensity was induced by MBL-mediated hydrolysis of CPC-1. It involved the incubation of VIM-27, NDM-1 and IMP-1 with the CPC-1 probe in the presence or absence of EDTA or phenylboronic acid (PBA). EDTA, a MBL inhibitor, significantly reduced the fluorescence signal in all MBL-treated samples, whereas PBA (an SBL inhibitor) had no effect. Additionally, the CPC-1 probe was incubated for 2 h with live MBL-producing *E. coli* strains (VIM-27 and IMP-1), a TEM-1-producing strain and a β-lactamase-negative control strain. Fluorescence imaging confirmed the selective detection of MBL-producing strains. Testing with clinical strains of MBL-producing *K. pneumoniae* and TEM-1-producing *E. coli* demonstrated that the CPC-1 probe can reliably distinguish clinical MBL-producers from non-producers. The lack of a lysis requirement makes the CPC-1 probe particularly well-suited for the selective imaging of live MBL-producing bacteria.

Song et al. [88] introduced CAT-7, an indirectly caged fluorogenic probe for the detection of MBLs. The synthesis of CAT-7 involved the conjugation of an arylated Tokyo Green (fluorescein derivative) fluorophore to a cephalosporin through a thiophenyl linker. Unlike direct caging strategies, in which the linker cages the fluorophore, this approach employs a distinct dinitrophenyl caging group. This modification confers highly efficient fluorescence quenching and enhances probe stability in aqueous solution. Activation of the probe by a β-lactamase results in the release of the thiophenyl linker. The linker subsequently induces an intramolecular substitution of the dinitrophenyl group, uncaging the fluorophore and producing a fluorescence signal. A longer linker length was correlated with a faster fluorescence response and greater stability when treated with β-lactamases. This factor was considered during the creation of an optimised CAT-7 probe. Following the exposure to a range of β-lactamases, CAT-7 demonstrated a high level of specificity toward MBLs (VIM-27, IMP-1 and NDM-1). Within 2 h, the probe exhibited a 77-fold increase in fluorescence intensity in the presence of IMP-1 (λ_ex/em = 490/510 nm). The incorporation of known MBL inhibitors (EDTA and captopril) successfully prevented the fluorescence signal activation, confirming the probe’s specificity. Subsequently, the CAT-7 probe was incubated with various bacterial lysates (4 × 10^5^ CFU in 100 μL PBS) for 2 h. Significant increases in fluorescence intensity were only observed amongst the clinical CRE strains (VIM-27, IMP-1 and NDM-1). Importantly, this caging strategy could be adapted to different fluorophores and employed for the detection of other analytes, thereby broadening its utility.

Ma and colleagues [89] introduced CARBA-H (Figure 2), an off-on dual colourimetric-fluorogenic probe for the detection of a broad spectrum of carbapenemases. The probe’s design incorporates a modified carbapenem scaffold, resorufin as a fluorophore, and a self-immolative allylic-hydroxybenzylic linker for broader carbapenemase recognition and higher probe sensitivity. The removal of 1β-methyl substituent from the carbapenem scaffold enables the probe to detect such carbapenemases as OXA-48, OXA-181 and OXA-232. Testing with 2 μM of the probe in PBS solution indicated that CARBA-H exhibits excellent sensitivity toward five clinically relevant carbapenemases (VIM-2, KPC-2, OXA-48, NDM-1 and IMP-1). Their LODs were reported to be in the picomolar (3.28 pM for VIM-2, 4.43 pM for KPC-2, 30.3 pM for OXA-48) or sub-picomolar (0.327 pM for NDM-1, 0.333 pM for IMP-1) range, within a 30-min timeframe (λ_ex/em = 564/629 nm). The probe was subsequently tested against a panel of 13 clinical CPE isolates (*K. pneumoniae* KPC-2, *E. coli* NDM-1, *E. coli* NDM-5, *E. coli* IMP-4, two strains of *E. coli* OXA-181, two strains of *K. pneumoniae* OXA-181, *E. coli* OXA-48, two strains of *E. aerogenes* OXA-48, *C. freundii* OXA-48, *K. pneumoniae* OXA-48) and 16 non-CPE control strains (eight isolates of *K. pneumoniae* and eight isolates of *E. coli*). The clinical isolates were lysed in a PBS buffer containing 0.5% CHAPS for 15 min prior to treatment with the probe. Following 15 min of incubation with CARBA-H, only the CPE strains displayed a significant increase in fluorescence intensity, detectable with a handheld UV lamp. Additionally, they produced a distinct chromogenic reaction visible under ambient light. Prolonged incubation (120 min) indicated that CARBA-H and resorufin are stable in complex biological environments. A parallel analysis of these strains with the CarbaNP test demonstrated the superior performance of CARBA-H over CarbaNP. The possibility of employing CARBA-H to detect carbapenemase activity in urine was subsequently investigated. Incubation of CPE-spiked urine samples with CARBA-H and 0.5% CHAPS for 2 h resulted in a significant increase in their fluorescence intensity (5 × 10^6^ CFU mL^−1^, 71 clinical isolates of CPE). These findings demonstrate the potential of CARBA-H for rapid and affordable POCT of CPOs.

Mao et al. [90] synthesised CB-1 (carbapenem-BODIPY), a fluorogenic probe with high selectivity toward carbapenemases. CB-1 is based on a carbapenem scaffold linked to an activatable BODIPY dye (alkenyl-linked boron dipyrromethene) fluorophore. Enzymatic activation by a carbapenemase results in a rapid >200-fold increase in fluorescence intensity (λ_ex/em = 500/535 nm). The strong green fluorescence of the probe reduces potential interference from the autofluorescence background of bacteria or the culture medium. After an incubation period of 30 min, the LOD of CB-1 was determined to be 1.1 pM for IMP-1, 7.7 pM for VIM-27, 2.7 pM for NDM-1 and 4.0 pM for KPC-3. The probe displayed only negligible changes in fluorescence intensity upon the introduction of several other β-lactamases (TEM-1, TEM-3, CTX-M-9), even at a concentration of 100 nM. The probe’s specificity was confirmed with an inhibition assay using EDTA and PBA. EDTA significantly decreased the fluorescence signal of the metal-dependent carbapenemases (IMP-1, NDM-1 and VIM-27), while PBA only affected the serine carbapenemase KPC-3. The high specificity of CB-1 toward carbapenemases was further tested by incubating it with several strains of *E. coli* which were transformed with carbapenemase genes (VIM-27, IMP-1, KPC-3). Strains of *E. coli* expressing TEM-1 and β-lactamase-negative *E. coli* were used as negative controls. Significant increases in fluorescence intensity were observed only in the carbapenemase-expressing strains. Furthermore, four CPO strains (NDM-1 *K. pneumoniae* ATCC BAA2146; VIM-1 *K. pneumoniae* NCTC 13440; OXA-48 *K. pneumoniae* NCTC 13442; MDR *A. baumannii* ATCC BAA1605), four non-CPO antibiotic-resistant strains (TEM-1 *E. coli* ATCC 35218; TEM-3 *E. coli* NCTC 13351; CTX-M-9 *E. cloacae* NCTC 13464; SHV-18 *K. pneumoniae* ATCC 700603) and an antibiotic-susceptible *E. coli* (LMG194) were incubated with CB-1 for 2 h. Only the CPOs produced a substantial fluorescence signal. These results indicate that CB-1 has excellent specificity for CPO detection, supporting its potential application in CPO screening. The probe’s high fluorescence turn-on ratio is particularly advantageous for the detection of enzymes at low concentrations.

Kim et al. [91] evaluated the effectiveness of various carbapenem-based fluorogenic probes for the detection of CPOs. Each probe incorporated different active linkers and fluorophores. Amongst all the tested probes, probe 1b demonstrated the highest stability and selectivity toward carbapenemases. Probe 1b consists of a hydroxymethyl carbapenem derivative moiety, an umbelliferone fluorophore and a benzyl ether active linker. The probe’s specificity was evaluated against four clinically revalent carbapenemases (IMP-1, NDM-1, KPC-3 and OXA-48), with TEM-1 as a negative control. One hour of treatment with probe 1b resulted in a substantial increase in fluorescence intensity of all tested carbapenemases (λ_ex/em = 365/460 nm). NDM-1 exhibited a >150-fold increase, while TEM-1 induced no significant change. The probe’s ability to detect CPOs was assessed on 24 clinical CPE strains and 18 clinical carbapenem-susceptible Enterobacterales (CSE) isolates. It was subsequently compared with the results of the manual Carba NP test performed on the same strains. The probe exhibited 91.7% sensitivity for CPE, surpassing Carba NP at 79.2%. Both methods achieved 100% sensitivity for KPC and NDM-1 carbapenemases. For OXA-48, the probe exhibited 66.7% sensitivity, compared with 16.7% for Carba NP. Collectively, these findings demonstrate the excellent selectivity of probe 1b for CPE over CSE and emphasize its clinical potential for rapid and accurate detection of CPOs. Furthermore, the study indicates that active linkers significantly influence probe stability.

Seok and colleagues [92] evaluated the performance of their recently developed rapid fluorogenic assay (Fluore), by comparing it with three other phenotypic assays–the modified carbapenem inactivation method, the Carba NP test and the carbapenemase inhibition test. The Fluore assay utilises a carbapenem-specific fluorogenic probe 1. It consists of a carbapenem moiety linked to an umbelliferone fluorophore via a hydroxybenzyl ether linker. Selective hydrolysis of the β-lactam ring by a carbapenemase initiates a rapid cascade reaction, resulting in the release of hydroxybenzyl alcohol and the fluorescent anionic form of umbelliferone (λ_ex/em = 360/465 nm). The linker not only initiates this reaction but also promotes the probe’s binding with a diverse range of carbapenemase active sites, enabling broad detection of CPE. The Fluore assay can be performed using both bacterial colonies (Fluore-C) and bacterial pellets obtained directly from positive blood cultures (Fluore-Direct). The assay’s performance was evaluated and compared to other phenotypic methods using a panel of previously characterised Enterobacterales isolates, including 63 CPE isolates: 36 producing KPC, one producing GES, nine producing NDM, five producing VIM, one producing IMP, six producing OXA, two co-producing KPC and OXA, and one co-producing NDM and OXA. Additionally, 154 non-CPE isolates were used: 48 non-carbapenemase-producing CRE, 53 ESBL producers and 53 third-generation cephalosporin-susceptible non-producers. The fluorescence signal was measured over 50 min using a fluorometer. Both the Fluore-C and Fluore-Direct versions of the assay demonstrated excellent performance. The fluorescence signals of CPE were significantly stronger than those of non-CPE, with particularly high fluorescence intensity observed in MBL producers. The fluorescence signal for the KPC and OXA strains was sufficiently strong to clearly distinguish them from the non-CPE strains. The assay exhibited excellent sensitivity in detecting KPC producers (100%) and class B carbapenemases (NDM 100%, VIM 100%, IMP 100%). The sensitivity of the assay for GES producers was reported to be 50% with Fluore-C and 33% with Fluore-Direct, while the sensitivity for OXA producers was 67%. The Fluore assay exhibited high accuracy compared to the modified carbapenem inactivation method, Carba NP test and carbapenemase inhibition test, with results available within 90 min.

Peng et al. [93] designed DNBS-CSA (2-(2′,4′-dinitrobenzenesulfonyl) 5-chlorosalicylaldehyde azine, Figure 3), a fluorescent probe that combines AIE and excited-state intramolecular proton transfer (ESIPT) mechanisms for the indirect detection of β-lactamase activity. DNBS-CSA is initially non-fluorescent. Its ESIPT process is restricted due to a sulfonate group caging one of its hydroxyl groups. In addition, the nitro groups on the benzene sulfonate serve as effective fluorescence quenchers. In the presence of a β-lactamase and a cephalosporin substrate, the probe undergoes a three-step reaction sequence. Initially, the β-lactam ring of cefazolin is cleaved by a β-lactamase, releasing a secondary amine that triggers a spontaneous elimination reaction. This yields a thiol-containing intermediate. The intermediate then reacts with the sulfonate group of DNBS-CSA, uncaging the hydroxyl group and releasing the salicylaldehyde azine derivative (CSA). When both essential hydroxyl groups in CSA are free, ESIPT and AIE can occur, producing strong yellow fluorescence (λ_ex/em = 383/558 nm). The dual AIE/ESIPT properties improve the system’s sensitivity and enable β-lactamase detection both in solutions and on test papers. In an aqueous PBS solution (10 mM, pH 7.4, 37 °C; DNBS-CSA 150 μM; cefazolin 4.8 mM), the fluorescence intensity exhibited a linear correlation with β-lactamase concentrations ranging from 0–10 mU mL^−1^ within 5–8 min of β-lactamase addition. The LOD for β-lactamases was determined to be 0.5 mU mL^−1^. Subsequently, the performance of DNBS-CSA on test papers was evaluated. The test papers were dipped in the sample solutions containing different concentrations of β-lactamases, incubated at 80 °C for 20 min and dried for 30 min. The recorded changes in fluorescence intensity exhibited a linear relationship with β-lactamase concentrations ranging from 0–2 mU mL^−1^. Owing to the AIE property of CSA, the fluorescence changes were observable by naked eye under UV light. Moreover, the applicability of DNBS-CSA for β-lactamase detection in milk was investigated. Incubation in a 10% milk solution yielded recoveries of 80–90% with RSD ≤ 10%, demonstrating the probe’s suitability for analysing real samples. DNBS-CSA also exhibited excellent selectivity for β-lactamases over other biomolecules and metal ions (such as vitamin C, lysozyme, esterase, Mg^2+^ and Ca^2+^). Compared to conventional direct-linkage probes, this indirect approach simplifies probe synthesis and provides a versatile, affordable method that is potentially suitable for POCT.

Yan et al. [94] developed DIcou-DNBS, a coumarin-derived fluorescent probe for thiol detection and indirect recognition of β-lactamases. DIcou-DNBS utilises 2,4-dinitrobenzenesulfonyl (DNBS) as a recognition site for biothiols. A double bond was constructed between 7-hydroxycoumarin-4-acetic acid and 7-diethylaminocoumarin-3-carbaldehyde to obtain the DIcou-OH fluorescent reporter group. DIcou-OH is characterised by a red emission and a significant Stokes shift of 122 nm. It can indirectly monitor β-lactamase activity by detecting MMT, a thiol-containing intermediate formed during the enzymatic cleavage of the β-lactam ring. MMT undergoes nucleophilic substitution with the DNBS moiety of the probe, releasing DIcou-OH which then produces a fluorescence signal. The probe’s selectivity was evaluated against biothiols (Cys, Hcy, GSH), a range of amino acids, ions, anions, glucose and citric acid. A marked increase in fluorescence intensity was observed exclusively in the presence of biothiols. An investigation across a pH range of 5.0–9.0 demonstrated that DIcou-DNBS can selectively recognise biothiols under conditions resembling the internal biological environment. Incubation of the probe with cefazolin sodium and a β-lactamase resulted in a marked, concentration-dependent increase in fluorescence intensity (λ_ex/em = 365/576 nm). Under UV light, the solution emitted a vivid red glow that was visible to the naked eye. The LOD was reported to be 2.1 × 10^−5^ U mL^−1^. As the concentration of the β-lactamase increased and the incubation time was extended, the fluorescence intensity continuously increased, reaching a maximum at 150 s. Subsequent testing involved four additional cephalosporin antibiotics (cefmetazole sodium, cefamandole sodium, cefamandole nafate sodium and ceftiofur sodium), each possessing distinct structural characteristics. Upon introduction of β-lactamase, DIcou-DNBS exhibited markedly higher fluorescence intensity after incubation with cefamandole nafate sodium and cefamandole sodium than with cefmetazole sodium or ceftiofur sodium, indicating faster hydrolysis of the former substrates. Testing on bacterial strains demonstrated that all five antibiotics exerted a substantial inhibitory effect against *S. aureus* ATCC 29213. When the antibiotics were applied to *E. cloacae* ATCC 13047, the probe produced variable fluorescence signals, reflecting differences in their hydrolysis rates. Notably, only ceftiofur sodium retained significant antibacterial activity, which is consistent with the results of the previous hydrolytic assessment. The suitability of DIcou-DNBS for screening β-lactamase inhibitors was confirmed using clavulanic acid and tazobactam. The probe effectively distinguished between the two compounds, enabling the calculation of their respective half-maximal inhibitory concentrations (29.5 μM and 3.76 μM). These findings collectively establish DIcou-DNBS as a highly sensitive, indirect probe for β-lactamase detection. It could serve as an effective tool for AST, screening for potential β-lactamase inhibitors and evaluating novel antibiotics.

Tummala et al. [95] proposed the use of fluorescent mesoporous silica nanoparticles (FMSNPs) for the rapid and sensitive detection of β-lactamases. The fluorescence is generated by conjugating the pH-sensitive fluorescein isothiocyanate (FITC) to the surface of the nanoparticles, which are then encapsulated with penicillin G (PenG). Upon encountering a β-lactamase, penicillin G is hydrolysed into penicilloic acid. The subsequent shift to an acidic environment causes fluorescence quenching of the particles (λ_ex/em = 490/530 nm). The performance of the probe was determined by incubating it with β-lactamase solutions with activities ranging from 0.5–0.00078 U mL^−1^. As long as β-lactamase activity exceeded 0.025 U mL^−1^, the relative fluorescence intensity of the probe decreased to approximately 75% within 1 h. The LOD for β-lactamases has been reported to be 7.8 × 10^−4^ U mL^−1^. In the absence of a β-lactamase, the fluorescence of FMSNP/PenG remained stable, retaining 99% of its initial intensity after 24 h of incubation. In addition, the FMSNP/PenG probe was tested on 25 clinical isolates which included both resistant and susceptible strains of *A. baumannii*, *K. pneumoniae* and *E. coli* to evaluate its clinical applicability. After incubating each bacterial suspension with FMSNP/PenG, the pH was monitored over time. Four strains exhibited no significant pH change, indicating a lack of β-lactamase production. These strains were also confirmed to be antibiotic-susceptible by a conventional testing method. The remaining 21 strains exhibited varying degrees of pH change, corresponding to different levels of β-lactamase activity. The changes in fluorescence intensity were subsequently compared between the resistant strains and the β-lactamase standards. All resistant strains yielded results that closely matched those of the standards, demonstrating the high sensitivity of the proposed method. Additionally, the performance of FMSNP/PenG was compared to that of a conventional acidimetric assay across a range of β-lactamase activities. The FMSNP/PenG assay was 2–3 times faster than the traditional method at equivalent β-lactamase activities. At a concentration of 0.5 U mL^−1^, the detection times were 30 min for FMSNP/PenG and 60 min for the acidimetric method. At a concentration of 0.00625 U mL^−1^, the respective detection times were 100 and 280 min. FMSNP/PenG also allows simultaneous detection and estimation of β-lactamase activity using the matching plots method. Moreover, the incorporation of mesoporous silica nanoparticles significantly enhanced the sensor’s stability.

Gong et al. [96] developed a colourimetric and ratiometric fluorescent nanoprobe (DPS NP) for the selective detection of H_2_S-associated β-lactam resistance. The nanoprobes self-assemble from an amphiphilic DPS dye, (E)-3-(3,3-dimethyl-2-styryl-3H-benzo[f]indol-1-ium-1-yl) propane-1-sulfonate. Within 5 min of the nucleophilic addition reaction with H_2_S, DPS NP exhibits a ratiometric fluorescence change (λ_ex = 435 nm) from yellow (553 nm) to blue (464 nm). The simultaneous yellow colourimetric response is also directly proportional to the amount of produced H_2_S. The LOD of DPS NP for H_2_S was determined to be 130 nM. The high biocompatibility of DPS NP was confirmed through an in vivo toxicity assessment on mice. The effect of antibiotics on endogenous H_2_S production in bacteria was investigated using antibiotic-susceptible and methicillin-resistant (Mu50) *S. aureus* strains. Bacterial suspensions were treated with 1 mM of vancomycin or one of several β-lactam antibiotics (oxacillin, cefalexin and cefazolin) for 15 min. The suspensions were subsequently incubated with 20 μM of DPS NP at 37 °C for 5 min, followed by 5 min of centrifugation. H_2_S concentrations were determined based on the fluorescence intensity ratio (I464/I553) measured with a spectrofluorometer. MRSA supernatant treated with cefazolin exhibited the strongest blue fluorescence, corresponding to the highest H_2_S production (~24 μM), while oxacillin and cefalexin induced comparable H_2_S levels. Antibiotic-susceptible *S. aureus* and vancomycin-treated MRSA produced minimal amounts of H_2_S (1.38–2.59 μM), likely attributable to bacterial pathways independent of β-lactamase activity. To evaluate the potential use of DPS NP for fluorescence imaging, 200 μL of DPS NP-treated bacterial suspensions were transferred to a 24-well plate. The suspensions were then fixed with 1 mL of an ethanol-acetic acid solution to enhance staining of the bacterial cells. Following 5 min of incubation, the cells were washed three times with PBS and imaged using fluorescence microscopy. Examination of fluorescent images with an overlay of the blue and green channels revealed that MRSA treated with β-lactams exhibited blue fluorescence, indicative of a high H_2_S concentration. The antibiotic-susceptible *S. aureus* strain and vancomycin-treated MRSA exhibited green fluorescence, indicating a high proportion of unreacted DPS NP. These results suggest that DPS NP could be effectively used for fluorescence imaging of BLRB. It could serve as a valuable tool for colorimetric and ratiometric fluorescence monitoring of H_2_S production in bacteria. Moreover, it facilitates rapid and sensitive screening for β-lactam antibiotic resistance.

Ding et al. [97] developed an AI-assisted mobile health system (mHealth) for rapid, quantitative detection of β-lactamases. The platform integrates a broad-spectrum fluorogenic probe (B1), a three-dimensional microfluidic paper-based analytical device (3D μPAD) and a smartphone-based AI cloud. The cloud enhances the system’s data-processing capabilities and improves test accuracy by correcting errors caused by temperature and pH variations. The B1 probe consists of a fluorescein-derived fluorophore and a cephalotin moiety that functions as a β-lactamase substrate. The probe’s specificity for β-lactamases was confirmed by incubating it with a panel of analytes, including TEM-1, other enzymes, ions, amino acids, and various biomolecules. Its LOD for TEM-1 was determined to be 0.13 nmol L^−1^ (λ_ex/em = 365/530 nm). Further testing with AmpC (class C) and CTX-M-14 (class A ESBL) demonstrated the probe’s rapid 20-s response and its broad-spectrum β-lactamase detection capability. The mHealth system was subsequently used to quantify β-lactamase levels in antibiotic-susceptible and antibiotic-resistant strains across various bacterial concentrations. The susceptible strains comprised *E. coli* (ATCC 25922), *S. aureus* (ATCC 29213) and *K. pneumoniae* (ATCC 13883). The resistant strains included an ESBL-producing clinical *E. coli* isolate, MDR *E. cloacae* (ATCC 13047), two MRSA strains (USA300 and clinical isolate ZJU) and two clinical *K. pneumoniae* strains (XJTU and PKU). After 20 min of incubation at room temperature, the samples were exposed to a handheld UV lamp and recorded with a smartphone. The images were then analysed by the AI cloud. The Rel. G values of all the tested antibiotic-susceptible groups were approximately 1.1. In contrast, the Rel. G values of MRSA (USA300 and ZJU), ESBL *E. coli*, MDR *E. cloacae* and clinical strains of *K. pneumoniae* (XJTU and PKU) significantly exceeded 1.1. This indicates that the mHealth system is capable of distinguishing between β-lactam-susceptible and resistant bacterial strains. To assess its POCT capability, the mHealth system was tested on serum, sewage, and saliva inoculated with varying concentrations of antibiotic-resistant *S. aureus*, *E. cloacae*, and *K. pneumoniae*, respectively. The results closely matched those obtained with a commercial kit. The mHealth system was subsequently employed to quantify β-lactamases over multiple time points in mouse infection models. A peritonitis model was created by injecting *E. coli* (ATCC 25922) and an ESBL-producing *E. coli* strain into the abdomen of mice, while the skin wound infection model involved the introduction of *S. aureus* (ATCC 29213) and MRSA (ZJU) into full-thickness skin wounds. At a 10^7^ CFU mL^−1^ inoculum, ESBL-producing *E. coli* exhibited a significant increase in the bacterial count and β-lactamase level in ascitic fluid over 24 h, while MRSA showed similar increases in wound exudate over 48 h. In both models, inoculation with 10^5^ CFU mL^−1^ of the resistant strains resulted in a progressive decline in bacterial counts and the absence of detectable β-lactamases. For both control strains, introduction of 10^5^ or 10^7^ CFU mL^−1^ yielded bacterial counts comparable to those of the resistant strains, with no detectable β-lactamase activity. These findings demonstrate the clinical potential of the mHealth system for AI-assisted POCT. It provides a highly sensitive, rapid and affordable approach that could be accessible even in remote areas.

Overall, these studies demonstrate how far fluorescent probe design for the detection of β-lactamases and BLPB has progressed in a relatively short time. Simple fluorogenic substrates now coexist with highly optimised NIR, self-immobilizing and dual-modal probes, nanostructured sensors and AI-assisted platforms, covering applications from rapid in vitro screening through ex vivo matrices to in vivo imaging in animal models. At the same time, several practical issues still limit routine clinical use, including probe specificity across diverse enzyme variants, stability and background in complex samples, manufacturing costs, as well as questions of biocompatibility and environmental impact. Future work will need to balance synthetic sophistication with robustness, affordability and ease of use, so that these fluorescent tools can move beyond proof-of-concept studies and become part of standard workflows for AST and resistance surveillance.

## 6. Conclusions

Over the past decade, luminescence-based assays employing fluorescent, chemiluminescent, and bioluminescent readouts have convincingly demonstrated that β-lactamase activity can be detected rapidly, sensitively, and in many cases without reliance on complex instrumentation. Across a wide spectrum of probe structures and assay formats, a consistent conclusion emerges luminescent approaches enable direct, activity-based interrogation of resistance mechanisms and, in several cases, allow functional discrimination between major β-lactamase classes based on substrate processing rather than gene presence alone. This functional dimension is particularly valuable for ESBLs and carbapenemases, where newly emerging variants often exhibit atypical or unpredictable activity profiles that may not be reliably inferred from genotypic data alone. At the same time, the body of literature reviewed here highlights important limitations that currently constrain broader clinical translation. Direct comparison of analytical performance across studies remains challenging due to substantial heterogeneity in luminescent platforms, assay designs, resistance mechanisms, and reported performance metrics. Limits of detection are variously expressed as enzyme activity, enzyme concentration, or bacterial load (CFU/mL), reflecting fundamentally different analytical targets that are not directly interchangeable. In addition, each luminescence modality carries inherent technical constraints, including autofluorescence and matrix-dependent signal interference in fluorescence-based assays, reagent complexity and intermediate instability in chemiluminescent systems, and reliance on engineered reporter strains or specific luciferase–luciferin pairs in bioluminescence-based approaches.

Common challenges across luminescent platforms also include variability in β-lactamase expression levels between bacterial isolates, which can significantly influence assay sensitivity, as well as the difficulty of resolving closely related enzyme variants with overlapping substrate profiles. Matrix effects in complex biological samples, limited validation on large and diverse clinical isolate collections, and practical considerations such as reagent stability, cost, and portability further complicate routine implementation, particularly in point-of-care settings. Despite these limitations, recent advances point to several realistic and promising directions for further development. These include the design of more stable and matrix-tolerant probes, the use of self-immobilizing or signal-amplifying chemistries to improve signal localization and robustness, and the simplification of assay workflows to reduce hands-on time and technical complexity. Emerging strategies such as multiplexed luminescent panels, nanomaterial-assisted sensing, and AI-supported data interpretation may further enhance diagnostic value by enabling parallel assessment of multiple resistance mechanisms within a single assay framework. If such technological progress is coupled with rigorous benchmarking against established phenotypic susceptibility tests and validation under clinically relevant conditions, luminescent β-lactamase assays have credible potential to become a practical complement to routine antimicrobial susceptibility testing. In this role, they would not replace conventional methods but rather provide rapid, functional information precisely where standard culture-based approaches are slowest, thereby supporting earlier and more informed therapeutic decision-making [98,99,100,101].

## Figures and Tables

**Table 1:** Functional and molecular β-lactamase classification schemes.

Bush-Jacoby-Medeiros Functional Classification	Ambler Molecular Classificiation	Catalytic Center	Substrate Hydrolysis Profile	Inhibition Profile	
				Tazobactam/Clavulanic Acid	EDTA
Group 1	Class C	Serine	Cephalosporins	No/Poor	No
Group 2	Class A and D	Serine	Diverse range (e.g., penicillins, cephalosporins, monobactams, carbapenems, carbenicillin, cefepime, cloxacillin)	Variable	No
Group 3	Class B	Zinc	Carbapenems	No	Yes

**Table 2:** Comparison of bioluminescence, chemiluminescence, and fluorescence in microbial applications.

Feature	Bioluminescence	Chemiluminescence	Fluorescence
Mechanism	Luciferase-catalysed oxidation	Light-emitting chemical reaction	Photon absorption and emission
External Excitation	Not required	Not required	Required
Typical Reporters/Substrates	lux, luc, coelenterazine systems	Luminol, acridinium esters, dioxetanes	Fluorescent dyes, GFP/mCherry, QDs
Signal Duration	Sustained (with substrate)	Short, transient (ms–min)	Variable (µs–s; some delayed)
Background Noise	Very low	Very low	Medium–high (autofluorescence)
Instrumentation	Luminometer	Luminometer	Fluorescence reader/microscope
Strengths	High sensitivity; ideal for in vivo imaging	Ultra-high sensitivity; robust enzyme assays	High spatial/temporal resolution
Use in Antibiotic Studies	Viability tracking, β-lactamase reporters	AMR detection, ROS assays	Cell integrity, single-cell dynamics, reporter strains

**Table 3:** Comparison of representative luminescence-based assays for phenotypic detection of β-lactamase activity and β-lactam resistance in bacteria. Reported LODs and assay times are reproduced as stated by the original authors and should be interpreted in the context of individual assay formats, target analytes (enzyme activity versus bacterial load), and experimental conditions. Where a classical LOD was not explicitly reported, performance is described using qualitative signal-to-noise ratios or classification accuracy. All assays were evaluated in vitro or ex vivo using purified enzymes, bacterial cultures, or clinical isolates.

Luminescence Type	Detection Probe	Identified Enzyme	Antibiotic Substrate	Source	Sample Preparation	LOD	Time	Ref.
Bioluminescence	D-Bluco (cephalosporin-caged D-luciferin with DABCYL quencher)	TEM-1, KPC-3, IMP-1, BlaC, AmpC, OXA-48	Cephalosporin (caged luciferin)	*E. coli*, *K. pneumoniae*, *E. cloacae* (laboratory strains and clinical isolates)	0.5% CHAPS lysis (15 min) + probe incubation (15 min); luciferase/ATP readout	0.01–0.1 fmol (enzymes); 10^2^–10^3^ CFU/mL (bacteria)	≤30 min (RAPID-BLI)	[52]
Bioluminescence	BacTiter-Glo (ATP-dependent luciferase assay)	Carbapenemases (KPC, NDM, VIM, IMP, OXA); non-CP (AmpC + porin loss)	Meropenem	CRE and CP-CRA (clinical isolates)	Colony suspension in MH/AST broth; incubation ± meropenem; lysis + BacTiter-Glo	Not reported (phenotypic cutoff)	~150 min	[53]
Chemiluminescence	Cephalosporin–dioxetane conjugate (CCP)	TEM-1, CTX-M-15, NDM-1, OXA-1	Cephalosporin	*E. coli* (clinical UTI isolates)	Purified enzyme or bacterial lysates; direct probe incubation	200 fM (TEM-1); 10^5^ CFU/mL (bacteria)	30–60 min	[59]
ECL	Ru(II)–ampicillin conjugate immobilized on TA@AuNPs/CNTs–Nafion electrode	β-lactamase (ampicillin- hydrolyzing)	Ampicillin	Purified enzyme	In vitro enzyme assay in PBS; electrode-based ECL readout	37 pg/mL (enzyme)	Not reported	[72]
Chemiluminescence	H_2_S-responsive dioxetane probes (probe 1 optimal)	βLBC, KPC-1/2, NMCA, SPM-1, Bla-1	Sulfur-containing β-lactams (e.g., sulopenem, cefazolin)	*K. pneumoniae*, *E. coli* (ATCC strain and clinical isolates)	Whole-cell incubation with antibiotic + probe (no lysis)	Not reported (S/N-based)	15–30 min	[73]
Chemiluminescence	CPCL (carbapenem–phenoxy-dioxetane probe with self-immolative linker)	Carbapenemases (SPM-1, VIM-15, KPC-1, NMCA)	Carbapenem	Purified enzymes, *P. aeruginosa*, *K. pneumoniae* (laboratory strains)	Whole colonies or broth suspensions mixed directly with probe (PBS, pH 7.4)	0.06 mU/mL (SPM-1); ~10^7^ CFU/mL (bacteria)	<10 min	[74]
Chemiluminescence	Array of enzyme-responsive phenoxy-dioxetane probes (12-probe CL panel)	Multiple enzymes incl. β-lactamase, phosphatase, β-glucuronidase, etc.	Penicillin-G derivative (for β-lactamase probe)	*S. pyogenes*, *S. mutans*, *S. aureus*, *S. epidermidis*, *B. cereus*, *B. subtilis*, *B. thuringiensis*, *E. faecalis*, *E. faecium*, *L. monocytogenes*, *E. coli*, *P. aeruginosa*, *K. pneumoniae*, *A. baumannii*, *H. influenzae B. cepacia*, *E. cloacae* (ATCC strains and clinical isolates)	Intact cells incubated with probe array in PBS (pH 7.4, 37 °C)	Not reported (classification accuracy-based)	~60–90 min	[75]
Chemiluminescence	HS-CL (H_2_S-activated phenoxy-dioxetane probe)	β-lactamase (via H_2_S generation)	Sulfur-containing β-lactams (e.g., ceftizoxime, cefalexin)	*E. coli*, *K. pneumoniae*, *P. aeruginosa*, *A. baumannii* (ATCC strains and clinical isolates)	Pre-incubation with antibiotic (3 h), followed by probe addition	1.02 μM (H_2_S); 10^2^–10^3^ CFU/mL (bacteria)	~180–210 min	[76]
Chemiluminescence	Relay CL system (CL-1 dioxetane + thiophenol-caged β-lactams BL-1/2/3)	β-lactamases (TEM-1, CTX-M-9, KPC-2, NDM-1/4/12, VIM-1, IMP-1, AmpC, OXA-1, MBLs)	Cephalosporins (1st/3rd gen) and carbapenem analogues	*E. coli*, *E. cloacae*, *K. pneumoniae* (laboratory strains and clinical isolates)	Whole-cell suspensions incubated with BL-n + CL-1 in 96-well plates	0.0049–0.026 nM (enzymes); ~10^7^ CFU/mL (bacteria)	~6 min	[77]
Fluorescence	CDA	Broad spectrum of β-lactamases	Cephalosporin	*E. coli*, *K. pneumoniae*, *E. cloacae*, *S. marcescens* (laboratory strains and clinical isolates)	Urine sample filtration/Live bacteria suspension, washing, CDA/H_2_O_2_ assay	10^3^ CFU mL^−1^ (bacteria, CDA/H_2_O_2_ assay)	120 min	[78]
Fluorescence	CDG-1	Broad spectrum of β-lactamases	Cephalosporin	Purified enzymes	None required (milk)	10^−3^ U mL^−1^ (TEM-1 or cephalosporinase; without FIMS); 10^−5^ U mL^−1^ (TEM-1; with FIMS)	15 min (probe); 30 min (probe with FIMS)	[79]
Fluorescence	CyPA-L	Broad spectrum of β-lactamases	Cephalosporin intermediate	*E. coli*, *K. pneumoniae*, *P. aeruginosa*, *S. aureus*, *E. faecalis* (laboratory strains and clinical isolates)	Liquid culture, centrifugation, washing, lysis of bacterial suspension, supernatant extraction	2.1 nM (TEM-1); 3.1 × 10^5^ CFU mL^−1^ (bacteria)	40 min	[80]
Fluorescence	BIN-3	Broad spectrum of β-lactamases	Cephalosporin	*E. coli*, *E. cloacae* (laboratory strains, in vivo mouse model)	Live bacteria suspension. In vivo (animal model); direct intravenous probe injection	2 × 10^6^ CFU (bacteria)	60 min	[81]
Fluorescence	CFC-2	Broad spectrum of β-lactamases	Cephalosporin intermediate	*E. coli*, *K. pneumoniae*, *A. baumannii* (ATCC/NCTC and laboratory strains)	Live bacteria suspension, incubation with the probe, washing	0.5 nM (TEM-1, lowest tested)	120 min	[82]
Fluorescence	CDC-559	AmpC β-lactamase	3-cephem-4-carboxylate (cephalosporin derivative)	*E. cloacae*, *S. aureus* (ATCC strains)	Liquid colony, dilution, uniform culture on solid medium	0.377 U mL^−1^ (enzyme)	30 min	[83]
Fluorescence	DFD-1	AmpC β-lactamase	Cephalosporin	*E. cloacae*, *P. aeruginosa*, *E. faecium*, *S. aureus*, *P. putida* (ATCC and laboratory strains)	Live bacteria suspension, incubation with LA-12, washing	Not reported	90 min	[84]
Fluorescence	RLB-2	Serine β-lactamases	Not reported	*E. cloacae*, *E. coli* (ATCC and laboratory strains)	Live bacteria suspension; Lysis of bacterial suspension, supernatant extraction	Not reported	180 min (bacteria)	[85]
Fluorescence	ERM-1	AmpC β-lactamase	Cephalosporin	*E. cloacae*, *E. coli* (ATCC and laboratory strains)	Live bacteria suspension	Not reported	60 min	[86]
Fluorescence	CPC-1	Metallo-β-lactamases	Carbapenem	*E. coli*, *K. pneumoniae* (laboratory strains and clinical isolates)	Live bacteria suspension	31 pM (IMP-1); 11 pM (VIM-27); 3 pM (NDM-1)	45 min (enzymes); 120 min (bacteria)	[87]
Fluorescence	CAT-7	Metallo-β-lactamases	Cephalosporin	*K. pneumoniae*, *E. coli* (clinical isolates)	Lysis of bacterial suspension, supernatant extraction	4 × 10^5^ CFU (bacteria, 100 mL, lowest tested)	120 min	[88]
Fluorescence	CARBA-H	Carbapenemases	Carbapenem derivative	*K. pneumoniae*, *E. coli*, *E. aerogenes*, *C. freundii* (clinical isolates)	Live bacteria suspension; Lysis of bacterial suspension, supernatant extraction	3.28 pM (VIM-2); 4.43 pM (KPC-2); 30.3 pM (OXA-48); 0.327 pM (NDM-1); 0.333 pM (IMP-1)	30 min	[89]
Fluorescence	CB-1	Carbapenemases	Carbapenem	*E. coli*, *K. pneumoniae*, *E. cloacae*, *A. baumannii* (ATCC/NCTC and laboratory strains)	Live bacteria suspension	1.1 pM (IMP-1); 7.7 pM (VIM-27); 2.7 pM (NDM-1); 4.0 pM (KPC-3)	30 min (enzymes);120 min (bacteria)	[90]
Fluorescence	1b	Carbapenemases	Carbapenem derivative	*E. coli*, *K. pneumoniae* (clinical isolates)	Lysis of bacterial suspension, supernatant extraction	10 nM (enzyme, lowest tested)	60 min	[91]
Fluorescence	Fluore assay probe 1	Carbapenemases	Carbapenem	Enterobacterales (CPE and non-CPE clinical isolates)	Blood culture, washing, pellet extraction, lysis of bacteria, supernatant extraction, dillution in PBS	1.5 × 10^6^ CFU/mL (bacteria, lowest tested)	90 min	[92]
Fluorescence	DNBS-CSA	Broad spectrum of β-lactamases–indirectly (detection of a thiol-containing intermediate)	Cephalosporin (added separately)	Purified enzymes and β-lactamase-spiked milk samples)	Not reported	0.5 mU mL^−1^ (enzyme)	5–8 min	[93]
Fluorescence	DIcou-DNBS	Broad spectrum of β-lactamases–indirectly (detection of a thiol-containing intermediate)	Cephalosporin (added separately)	*E. cloacae*, *S. aureus* (ATCC strains)	TSB bacteria culture, dillution, uniform culture on MH agar	2.1 × 10^−5^ U mL^−1^ (enzyme)	2.5 min	[94]
Fluorescence	FMSNP/PenG	Broad spectrum of β-lactamases	Penicillin G	*A. baumannii*, *K. pneumoniae*, *E. coli* (clinical isolates)	Live bacterial suspension	7.8 × 10^−4^ U mL^−1^ (enzyme)	30 min	[95]
Fluorescence	DPS NP	Broad spectrum of β-lactamases–indirectly through the detection of H_2_S	Vancomycin/Oxacillin/Cefalexin/Cefazolin	*S. aureus* (antibiotic-susceptible and MRSA laboratory strains)	Live bacterial suspension, treatment with an antibiotic, fixing, incubation with the probe, washing	130 nM (H_2_S)	5 min	[96]
Fluorescence	B1	Broad spectrum of β-lactamases	Cephalotin	*E. coli*, *K. pneumoniae*, *E. cloacae*, *S. aureus* (ATCC and laboratory strains, clinical isolates, in vivo mouse models)	Direct loading of 3D μPAD with live bacteria suspension or bacterial lysate	0.13 nmol L^−1^ (TEM-1)	20 s (enzyme)	[97]

Abbreviations: CRE—Carbapenem-resistant Enterobacterales; CP—carbapenemase-producing; CRA—carbapenem-resistant *Acinetobacter*; UTI—urinary tract infection; MH—Mueller Hinton; AST—antibiotic susceptibility testing; ECL—electrogenerated chemiluminescence; PBS—phosphate buffered saline; CPE—carbapenemase-producing Enterobacterales; MRSA—methicillin-resistant *Staphylococcus aureus*. Note: Reported limits of detection (LOD) reflect different analytical targets depending on the assay design, including purified enzyme activity, bacterial lysates, or viable bacterial counts (CFU/mL). These values should therefore be interpreted within the context of each platform rather than compared directly across methods.

## Data Availability

No new data were created or analyzed in this study. Data sharing is not applicable to this article.

## Abbreviations

The following abbreviations are used in this manuscript:

AIE	Aggregation-induced emission
AMR	Antimicrobial resistance
AST	Antibiotic susceptibility testing
BLPB	β-lactamase-producing bacteria
BLRB	β-lactam-resistant bacteria
BRET	Bioluminescence resonance energy transfer
BSA	Bovine serum albumin
CFU	Colony-forming unit
CIEEL	Chemically initiated electron exchange luminescence
CLEIA	Chemiluminescent enzyme immunoassay
CP	Carbapenemase-producing
CPE	Carbapenemase-producing Enterobacterales
CRA	Carbapenem-resistant *Acinetobacter*
CRE	Carbapenem-resistant Enterobacterales
CPO	Carbapenemase-producing organisms
CSE	Carbapenem-susceptible Enterobacterales
DABCYL	4-(4-dimethylaminophenylazo)benzoic acid
DBCO	Dibenzocyclooctyne
ECL	Electrogenerated chemiluminescence
ESBL	Extended-spectrum β-lactamases
FRET	Fluorescence resonance energy transfer
LOD	Limit of detection
MBL	Metallo-β-lactamase
MDR	Multidrug-resistant
MH	Mueller Hinton
MIC	Minimal inhibitory concentration
MRSA	Methicillin-resistant *S. aureus*
NIR	Near-infrared
PBA	Phenylboronic acid
PBS	Phosphate-buffered saline
PCR	Polymerase chain reaction
POC	Point-of-care
POCT	Point-of-care-testing
ROS	Reactive oxygen species
SBL	Serine β-lactamase
TPE	Tetraphenylethylene
UTI	Urinary tract infection
