# Correction: Korytkowski et al. Luminescence-Based Strategies for Detecting β-Lactamase Activity: A Review of the Last Decade. *Life* 2026, *16*, 250

**DOI:** 10.3390/life16050798

**Published:** 2026-05-11

**Authors:** Michał Jakub Korytkowski, Anna Baraniak, Alicja Boryło, Paweł Rudnicki-Velasquez

**Affiliations:** 1 Department of Pharmaceutical Microbiology and Laboratory Diagnostics, National Medicines Institute, Chełmska 30/34, 00-725 Warsaw, Poland; m.korytkowski@nil.gov.pl (M.J.K.);; 2 Department of Chemistry and Radiochemistry of Environment, Faculty of Chemistry, University of Gdansk, Wita Stwosza 63 Str., 80-308 Gdansk, Poland; 3 Department of Falsified Medicines and Medical Devices, National Medicines Institute, Chełmska 30/34, 00-725 Warsaw, Poland

In the original publication [1], there is an error about the entry of Substrate Hydrolysis Profile for Group 3 (Ambler class B). The corrected Table 1 appears below. The authors state that the scientific conclusions are unaffected. This correction was approved by the Academic Editor. The original publication has also been updated.

## Figures and Tables

**Table 1:** Functional and molecular β-lactamase classification schemes.

Bush-Jacoby-Medeiros Functional Classification	Ambler Molecular Classification	Catalytic Center	Substrate Hydrolysis Profile	Inhibition Profile	
				Tazobactam/Clavulanic Acid	EDTA
Group 1	Class C	Serine	Cephalosporins	No/Poor	No
Group 2	Class A and D	Serine	Diverse range (e.g., penicillins, cephalosporins, monobactams, carbapenems, carbenicillin, cefepime, cloxacillin)	Variable	No
Group 3	Class B	Zinc	Penicillins, cephalosporins, carbapenems	No	Yes
